# Vaccinia Virus—A Swiss Army Knife Against Cancer

**DOI:** 10.3390/cancers17142324

**Published:** 2025-07-12

**Authors:** Marcin Stawowczyk, Yanqi Ye, Nanhai G. Chen

**Affiliations:** ViroMissile, Inc., 505 Coast Blvd South, Suite 208, La Jolla, CA 92037, USA

**Keywords:** vaccinia, oncolytic virus, cancer, virotherapy, immunotherapy, oncology

## Abstract

Despite significant advances in cancer therapy, the prognosis for patients with advanced, disseminated disease remains poor. This underscores the urgent need for novel treatments that not only eliminate tumor cells effectively but also stimulate a strong, durable anti-cancer immune response. Among emerging strategies, oncolytic viruses have shown exceptional promise due to their selective cytotoxicity and their ability to activate T cell-mediated immune responses. In this review, we focus on the vaccinia virus (VACV), a member of the *Poxviridae* family, which has emerged as a leading candidate in modern oncolytic immunotherapy. We examine the virus’s properties that enable it to evade antiviral defenses and serve as a versatile, potent oncolytic agent. Furthermore, we explore its interactions with various components of the immune system and how these contribute to the induction of a robust T cell-driven response. Finally, we assess current efforts to harness VACV for the treatment of various cancer types and highlight future directions where its application is most likely to succeed. Overall, our goal is to present VACV as a powerful and broadly applicable platform with the potential to transform the landscape of oncology.

## Simple Summary

This review explores the potential of the vaccinia virus (VACV) as a powerful tool in oncolytic cancer treatment. While current therapies often fall short for patients with advanced cancer, VACV offers a promising approach by directly killing tumor cells and activating the body’s immune system—particularly T cells—to fight cancer. The review highlights the virus’s unique ability to evade immune defenses, its interactions with the immune system, and ongoing research into its use as an oncolytic agent against various cancer types. Overall, VACV is presented as a versatile and impactful candidate for future cancer therapies.

## 1. Introduction

Vaccinia virus (VACV) is an *Orthopoxvirus*, and a prototypic member of large *Poxviridae* family. Due to its genomic and antigenic similarities to variola virus, along with high immunogenicity and low virulence, VACV was extensively used as a smallpox vaccine, ultimately contributing to the successful eradication of the disease. Like other *Orthopoxviruses*, it replicates exclusively in the cytoplasm and does not integrate into the host genome [1,2,3]. The exact origin of VACV remains unclear: One theory suggests that it evolved from either smallpox or cowpox virus; another posits that all three viruses share a common ancestor; a third proposes that it may have been derived from horsepox virus [4]. In the wild, VACV has been reported to cause sporadic infections in cattle and water buffalos, but its natural host and reservoir remain unknown [5].

VACV is an enveloped, double-stranded DNA virus with a relatively large genome size of approximately 190 to 200 kb. Both DNA strands are connected at the termini by partially complementary AT-rich loops, forming one continuous polynucleotide chain [6]. Interestingly, despite many years of genomic and transcriptomic research, the exact number of genes and proteins encoded by the VACV genome remains unknown. Most sources estimate the number of VACV genes to be between 200 and 250 [7,8,9].

Over the years, several strains of VACV have been developed. The most studied strain is the Western Reserve (WR) strain, which was created in the United States through repeated passages of the New York City Board of Health (NYCBH) strain in rabbits, mice, and various mammalian cell cultures. Due to its adaptation to multiple hosts, it produces high titer in vitro and causes neuropathogenic effects in vivo, making it unsuitable for use as a vaccine [10].

In contrast to the WR strain, the Lister strain was developed at the Lister Institute in the United Kingdom, and the NYCBH strain is much less virulent while still retaining the ability to replicate in humans and other mammals. The Lister and Wyeth strains (with Wyeth being the commercial name for the NYCBH strain) were most commonly used for vaccinations during the smallpox eradication campaign [11]. They were known to induce strong immunity accompanied by high antibody titers, although they occasionally caused undesired side effects, including severe eczema and encephalitis [12].

Other VACV strains include Copenhagen and IHD (International Health Department), the latter being particularly notable for high production of extracellular enveloped viruses (EEVs) [13]. Finally, the modified vaccinia virus Ankara (MVA) strain represents a more attenuated version of the VACV Ankara strain. MVA cannot replicate in vivo and exhibits only limited replication in mammalian cells in vitro. It was developed through over 570 serial passages of the original virus in primary chicken embryo fibroblasts, resulting in the deletion of over 30 kb fragment of its genome [11].

Today, most oncolytic VACV strains developed for therapy are based on various modifications of strains such as Lister, Wyeth, Copenhagen, WR, or IHD.

## 2. Infection and Replication

### 2.1. Viral Entry

**Figure 1:**
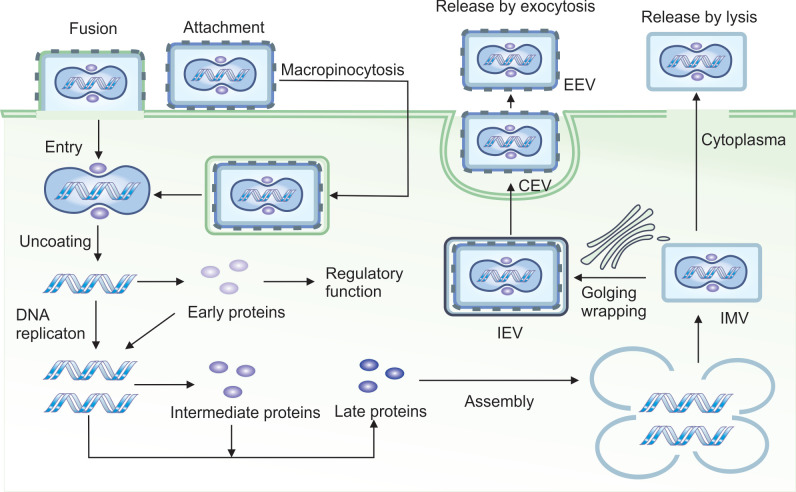
Life cycle of vaccinia virus. VACV enters host cells either through direct membrane fusion (primary route) or via macropinocytosis (secondary route) after binding to the cell surface. Following entry, the viral core is released into the cytoplasm, where core-associated enzymes initiate transcription of early genes. These early genes promote viral DNA replication and suppress host antiviral responses. DNA replication then triggers the expression of intermediate genes, which in turn activates the transcription of late genes responsible for virion assembly. VACV produces four distinct forms of virions: (1) intracellular mature virions (IMVs) that accumulate within the cytoplasm and are released only upon cell lysis; (2) intracellular enveloped virions (IEVs) that acquire additional membranes from the trans-Golgi network or endosomes and are transported the cell surface via microtubules; (3) cell-associated enveloped virions (CEVs) that are formed when IEVs fuse with the plasma membrane, enabling direct cell-to-cell spread; (4) extracellular enveloped virions (EEVs), a subset of CEVs that are released from the cell and facilitate long-range viral dissemination to distant cells.

A schematic illustration of the VACV’s life cycle is shown in Figure 1. In contrast to the majority of viruses such as adenoviruses or herpesviruses, VACV does not rely on binding to a specific cellular receptor for entry. Instead, it attaches to host cells using glycosaminoglycans (GAGs), specifically heparan sulfate and chondroitin sulfate for attachment [14,15]. Since GAGs are widely expressed on a variety of cell types, this broadens VACV’s host range, allowing it to infect multiple cell types across different species.

Additionally, a scavenger receptor MARCO (Macrophage receptor with collagenous structure) has been reported to facilitate VACV binding and enhance its interaction with GAGs [16].

The primary and most common mechanism of VACV entry involves direct fusion of the viral and cell membranes. This multistep process includes close apposition of both membranes, lipid mixing of the outer membrane leaflets to form a hemifusion intermediate, followed by the formation and expansion of a fusion pore, which allows the viral core or nucleoprotein to enter the cytoplasm [17]. Notably, this fusion can occur at neutral pH, allowing VACV to bypass the endocytic pathway. As a result, the virus avoids pathogen recognition receptors (PRRs) localized in endosomes and can initiate replication rapidly.

Alternatively, as a secondary route, VACV can enter cells via macropinocytosis, wherein membrane fusion occurs within endosomal vesicles and is triggered by lower pH [18]. This pathway is complex and involves Rho GTPases and the tyrosine kinase activity of the epidermal growth factor receptor (EGFR) in the host cell [19]. Depending on the strain, VACV may induce the formation of blebs or filopodia in the target cell to facilitate the internalization of viral particles.

Overall, at least 16 different viral proteins are involved in VACV entry: 4 in attachment and 12 in penetration. However, the precise molecular details of these processes remain incompletely understood [17].

### 2.2. Replication

The VACV virion consists of a compact protein core tightly wrapped around the viral DNA, along with two lateral bodies that contain proteins involved in counteracting host antiviral defenses [20]. Almost immediately after VACV enters a host cell, the lateral bodies dissociate from the core, leading to the release of H1 phosphatase, which inactivates the signal transducer and activates transcription 1 (STAT1) protein, thereby blocking interferon signaling [21].

Within 20 min of entry, components of the viral core initiate transcription of approximately 50% of the viral genome, producing a set of early mRNAs [22]. These early transcripts encode proteins required for DNA replication, which begins roughly 2 h post-infection [23]. DNA replication then triggers the expression of intermediate genes, followed by late genes essential for the assembly of mature virions [22]. The first complete virions are typically produced around 6 h after initial infection, and cell lysis generally begins within 15–48 h post-entry [24].

A hallmark of VACV-infected cells is the presence of cytoplasmic structures known as virosomes or virus factories. These complexes, composed of viral proteins and protrusions of endoplasmic reticulum (ER) and Golgi-derived membranes, are the sites of viral DNA replication, transcription, and virion assembly [22,25].

To carry out replication independently of the host cell nucleus, VACV encodes a broad array of viral replication machinery, including enzymes that substitute for nuclear counterparts. These include DNA polymerase (E9), uracil DNA glycosylase (D4), and D5 protein, which possesses helicase and primase activity [26]. Additional proteins such as single-strand DNA-binding protein (I3) [27], Holliday junction resolvase (A22) [28], and ligase (A50) [29] further support autonomous replication, thereby minimizing the risk of non-specific integration into the host genome.

VACV also synthesizes its own nucleotide precursors via enzymes such as thymidine kinase (TK; encoded by J2R) [30], thymidylate kinase (encoded by A48R) [31], and ribonucleotide reductase (RR; encoded by F4L and I4L) [32].

During its replication cycle, VACV produces four distinct types of virions: intracellular mature virus (IMV), intracellular enveloped virus (IEV), cell-associated enveloped virus (CEV), and extracellular enveloped virus (EEV) [33]. Different VACV strains produce varying proportions of virion types, which affects their spread, infectivity, and ability to evade the immune system [34]. IMV is the most abundant and stable form of the virus, remaining within the cell until released by lysis. It is well-suited for host-to-host transmission. IEV is formed when IMV is wrapped in a secondary membrane, typically derived from the Golgi apparatus, and is transported to the cell surface along microtubules for dissemination. After fusing with the plasma membrane, the IEV loses two of its envelope proteins and becomes a CEV, a form that remains attached to the cell surface [35]. CEV induces the formation of actin tails, which propel the virus away from the cell, facilitating cell-to-cell spread. EEV is the fully released form, enabling long-range dissemination of the virus [35].

It is estimated that approximately 2500–5000 new viral particles are produced per infected cell [36].

## 3. Strategies to Avoid Antiviral Mechanisms

Mammalian cells have evolved numerous strategies to detect viruses, block their replication, and inhibit their spread. The general antiviral mechanism involves recognition of the pathogen with specific receptors, signal transduction leading to activation of transcription factors, and expression of target genes that help resolve the infection and alert the neighboring cells.

Most antiviral genes are regulated by two key transcription factors: interferon regulatory factor 3 (IRF3) (along with its relatives IRF5 and IRF7) and Nuclear Factor-kappa B (NF-κB). IRF3 is essential for the induction of type I interferons (IFNs): IFN-α and IFN-β—cytokines secreted by infected cells that activate the expression of interferon-stimulated genes (ISGs) in the surrounding tissue. ISGs function to block viral replication, promote antigen presentation, and eliminate infected cells through apoptosis [37].

NF-κB, on the other hand, triggers the transcription of multiple pro-inflammatory cytokines (e.g., tumor necrosis factor (TNF) α, interleukins (IL) IL-1β, IL-6, IL-8) and chemokines (e.g., chemokine (C-X-C motif) ligand1 (CXCL1), CCL5, CCL9), which recruit and stimulate immune cells, induce fever, and enable a rapid and tailored response to the specific pathogen [38].

**Figure 2:**
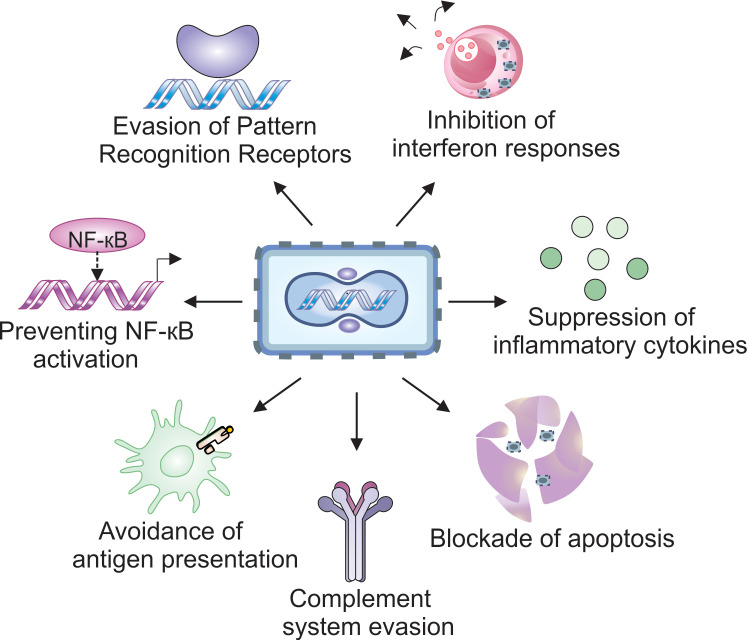
Inhibition of antiviral mechanisms by vaccinia virus. VACV has evolved multiple strategies to evade host immune responses at both molecular and cellular levels. It encodes multiple viral proteins that disrupt pathogen recognition receptor (PRR) signaling pathways, including those mediated by cGAS, DNA-PK, and Toll-like receptors (TLRs). These viral proteins inhibit key transcription factors such as interferon regulatory factor 3 (IRF3) and NF-κB, thereby blocking the expression of pro-inflammatory and antiviral genes. VACV also impairs interferon (IFN) signaling by suppressing IFN production, scavenging soluble IFNs, and blocking downstream signaling from IFN receptors. Additionally, the virus secretes immunomodulatory proteins that neutralize pro-inflammatory cytokines. To promote efficient viral replication, VACV encodes multiple proteins that inhibit apoptosis by targeting key components of apoptotic pathways. It also interferes with antigen presentation by antigen-presenting cells (APCs) and suppresses the activation of other immune cells, including natural killer (NK) cells and T cells. Finally, VACV expresses a distinct set of proteins that inhibit the complement cascade, further promoting immune evasion.

To ensure effective replication, VACV has evolved a range of mechanisms to inhibit the IRF3 and NF-κB pathways at multiple levels, including pathogen recognition, signal transduction, transcription factor activation, and cytokine production (see Figure 2). Below, we briefly discuss how various VACV-encoded proteins effectively disarm the host antiviral response.

### 3.1. DNA-PK and Viral DNA Detection

**Figure 3:**
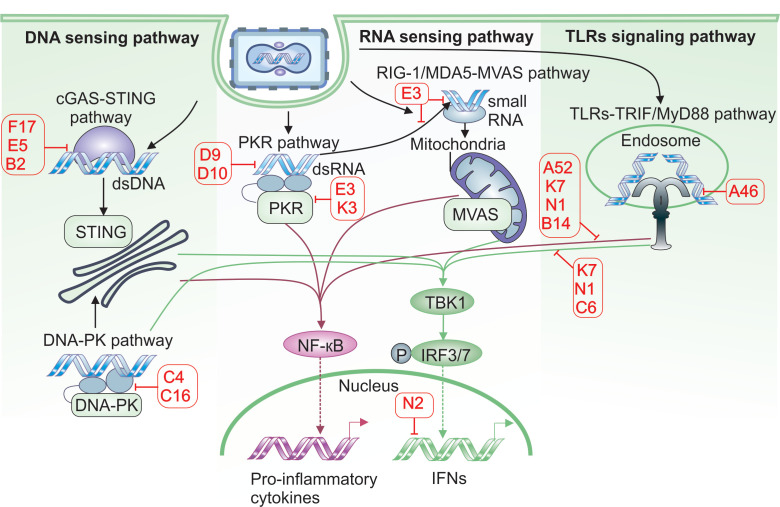
Antiviral sensing pathways and vaccinia virus antagonists. VACV encodes a wide array of proteins that antagonize host antiviral sensing mechanisms, including cytosolic DNA sensing, RNA sensing, and Toll-Like Receptors (TLRs) signaling pathways. In the cytosolic DNA sensing pathway, the Stimulator of Interferon Genes (STING) axis is inhibited by VACV proteins F17 and E5, which promote degradation of the DNA sensor Cyclic GMP-AMP Synthase (cGAS). Additionally, B2 degrades Cyclic GMP-AMP (cGAMP), thereby preventing the transmission of antiviral signals to neighboring cells. The DNA sensor DNA-dependent protein kinase (DNA-PK) is also targeted by C4 and C16, further impairing DNA-triggered immune responses. In the RNA sensing pathway, VACV proteins D9, D10, E3, and K3 inhibit activation of Protein Kinase R (PKR). E3 also binds and sequesters viral RNA, shielding it from recognition by Retinoic Acid-Inducible Gene I/Melanoma Differentiation-Associated Protein 5 (RIG-I/MDA5). TLR-mediated signaling is disrupted by multiple viral proteins: A46 blocks interactions between TLRs and adaptor proteins, preventing IRF3 activation; A52 inhibits NF-κB activation; and N1 impairs both IRF3 and NF-κB pathways. Key downstream signaling hubs are also targeted. The serine/threonine kinase TBK1, essential for IRF3 activation downstream of STING, RNA sensors, and TLRs, is inhibited by C6 and K7. In the nucleus, N2 functions as a direct IRF3 inhibitor, preventing transcription of interferon-stimulated genes.

As a DNA virus that replicates in the cytosol, VACV is particularly vulnerable to detection by PRRs, which activate signaling pathways involving IRFs, NF-κB, and adaptor proteins such as the stimulator of interferon genes (STING). To avoid being recognized by PRRs, VACV developed a number of sophisticated mechanisms that are briefly summarized in Figure 3.

One of VACV’s evasion strategies targets the DNA-dependent protein kinase (DNA-PK) complex, which senses cytosolic double-stranded DNA and activates IRF3-mediated IFN production through both STING-dependent and -independent pathways [39]. Two proteins, C16 and C4, encoded by two copies of the C16L gene and the C4L gene, respectively, bind to the Ku70/Ku80 heterodimer within the DNA-PK complex via their C-terminal domains. This interaction inhibits DNA-PK’s ability to detect cytosolic DNA [40,41]. Interestingly, C16 and C4 appear to function redundantly, as both bind to DNA-PK in a similar manner. This redundancy is believed to have evolved to counteract the high levels of DNA-PK present in certain cell types, such as fibroblasts, which are primary targets of VACV infection [39].

Another DNA-sensing mechanism involves DNA-dependent RNA polymerase III (Pol III). Although predominantly nuclear, Pol III is also detected in the cytosol, where it detects AT-rich DNA sequences. Upon recognizing viral DNA, Pol III synthesizes 5′-triphosphate-containing double-stranded (ds)RNA, which activates the RNA sensor Retinoic acid-inducible gene I (RIG-I), leading to downstream activation of IRF3 and NF-κB [42]. The VACV E3 protein inhibits Pol III activity, thereby blocking NF-κB activation. Although E3 contains both RNA- and DNA-binding domains, it is the RNA-binding domain that is responsible for inhibiting Pol III function [43]. VACV’s DNA can also be detected by IFN-γ-inducible protein 16 (IFI16), which, like Pol III, resides mainly in the nucleus but can also be found in the cytosol. IFI16 has been shown to activate both IRF3 and NF-κB in response to viral infections [44]. However, the VACV WR strain can inhibit IFI16-dependent expression of CCL5 and IFN-stimulated genes, although the precise mechanisms underlying this inhibition remain unclear [45].

### 3.2. cGAS, STING and IRF3

A distinct set of VACV proteins has evolved to counteract the cGAS-STING pathway, one of the most critical antiviral defense systems against cytosolic DNA viruses. Cyclic GMP-AMP synthase (cGAS) senses double-stranded DNA and produces 2′3′-cyclic GMP-AMP (cGAMP), a second messenger that activates the STING adaptor protein. This activation triggers TANK-binding kinase 1 (TBK1)-mediated phosphorylation of IRF3, leading to the induction of type I IFN responses.

Importantly, cGAMP’s activity is not confined to the infected cell. It can diffuse into neighboring cells through gap junctions and membrane fusion, or be actively imported from the extracellular milieu [46,47]. As a result, cGAMP produced in even a single cell can initiate a broader antiviral response in surrounding uninfected tissue, thereby limiting viral spread. To overcome this barrier, VACV has evolved multiple proteins that inhibit the cGAS-STING pathway at various levels.

One such protein is F17, which suppresses cGAS activity by hyperactivating the mammalian target of rapamycin (mTOR) pathway. cGAS is known to be inhibited by Akt kinase, which phosphorylates specific serine residues, thereby reducing its activity [48]. F17 promotes this effect by sequestering two key regulatory subunits of the mTOR complexes, Raptor and Rictor, leading to excessive mTOR activation. This, in turn, stimulates Akt signaling and enhances cGAS degradation [49]. In addition, hyperactive mTOR promotes increased protein synthesis, supporting efficient viral protein production and virion assembly [49].

Another key VACV protein, E5 (encoded by the E5R gene), binds directly to cGAS, inducing its polyubiquitination and subsequent proteasomal degradation. In the presence of E5, intracellular cGAS levels are reduced, resulting in strong inhibition of type I IFN induction [50]. E5 is supported by B2—also known as poxin and encoded by the B2R gene—which enzymatically cleaves cGAMP into an inactive linear form. Although B2 has little effect on VACV replication in vitro, its absence significantly reduces viral titers in mouse models, highlighting its importance for in vivo viral spread [51].

VACV also interferes with signaling downstream of STING by targeting the TBK1 complex. The viral protein C6 binds with the cellular TBK1 adaptor proteins TRAF family member-associated NF-kappa-B activator (TANK), similar to NAP1 TBK1 adaptor (SINTBAD), and NAK-associated protein 1 (NAP1), thereby preventing effective IRF3 phosphorylation [52]. Another protein, K7, blocks TBK1 activity by interacting with DEAD-box RNA helicase DDX3, an essential adaptor and substrate for optimal IRF3-mediated responses [53]. Finally, N2—a member of the VACV Bcl-2 family—acts in the nucleus where it inhibits IRF3’s ability to activate gene expression under the IFN-β promoter [54]. While deletion of any of these viral genes does not impair VACV replication in vitro, all significantly affect virulence in mouse models [52,53,54].

### 3.3. Double-Stranded RNA Sensors

Although VACV is a dsDNA virus, it generates significant amounts of dsRNA during its replication cycle due to the synthesis of overlapping transcripts that often anneal into duplexes [55]. As a result, VACV becomes a target for several cellular RNA sensors.

One such sensor is protein kinase R (PKR), which recognizes dsRNA and phosphorylates the eukaryotic translation initiation factor 2 (eIF2). This phosphorylation inhibits cap-dependent translation, ultimately leading to a shutdown of protein synthesis and induction of cell death [56]. To evade PKR-mediated antiviral responses, VACV expresses several proteins—encoded by E3L, K3L, D9R, and D10R—that directly or indirectly inhibit PKR function. The E3 protein binds directly to viral dsRNA, shielding it from PKR recognition and preventing its dimerization and activation [57]. The K3 protein mimics cellular eIF2 and acts as a decoy substrate for PKR, diverting phosphorylation away from the host’s eIF2 [58]. In addition, D9 and D10 function as decapping enzymes for dsRNA, reducing the stability and concentration of viral dsRNA, thereby lowering its visibility to PKR [59].

VACV also inhibits another key dsRNA-sensing pathway involving oligoadenylate synthases (OAS1, OAS2, and OAS3). Upon dsRNA detection, these enzymes produce 2′-5′-oligoadenylates (2–5 As), which activate RNAase L, leading to the degradation of cellular and viral mRNAs, shutdown of protein synthesis, and induction of apoptosis. The E3 protein sequesters dsRNA, preventing its recognition by OAS proteins and thereby blocking RNAase L activation [60]. Similarly, D9 and D10 proteins reduce dsRNA levels further limiting activation of the OAS/RNAase L pathway [59].

Two additional cytosolic sensors critical for type I IFN induction are RIG-I and melanoma differentiation-associated protein 5 (MDA-5) proteins. They both have helicase activity and can detect dsRNA in the cytosol of infected cells. RIG-I is more specific for shorter RNAs with triphosphate groups, while MDA-5 shows higher affinity for longer dsRNA [61]. Upon activation, both sensors interact with the mitochondrial adapter protein Mitochondrial Antiviral Signaling (MAVS), which activates TBK1, leading to IRF3 phosphorylation and induction of type I IFN genes. MAVS can also recruit TNF receptor (TNFR)–associated factor 6 (TRAF6), triggering activation of the NF-κB pathway [61].

VACV’s E3 protein has a potent inhibitory effect on the RIG-I/MDA5 activation. Studies have shown that expression of the E3 RNA-binding domain alone is sufficient to completely block cytokine induction downstream of RIG-I/MDA5 activation [62]. E3 masks viral dsRNA from recognition by these sensors and also inhibits RNA polymerase III, which generates 5′-triphosphate-containing dsRNA species that activate RIG-I [43]. Through these combined actions, VACV effectively suppresses both RNA- and DNA-dependent IFN activation pathways.

### 3.4. Toll-like Receptor System

Toll-like receptors (TLRs) are a family of 13 transmembrane PRRs localized on the plasma membrane and within intracellular compartments such as endosomes, ER, and lysosomes. TLRs are characterized by an N-terminal extracellular leucine-rich repeat-containing ectodomain, a single transmembrane helix, and a C-terminal cytoplasmic Toll/interleukin-1 receptor (TIR) domain. These receptors specialize in detecting pathogen-associated molecular patterns (PAMPs), such as viral nucleic acids, lipopolysaccharide (LPS) or flagellin [63].

Upon recognition of PAMPs, TLRs can signal through either the Myeloid differentiation primary response 88 (MyD88) adapter protein—activating NF-κB (e.g., TLRs 2, 6, 7, 8 and 9)—or through the TIR-domain-containing adaptor-inducing interferon-β (TRIF), used by TLR3 and TLR4, leading to IRF3 activation and the expression of type I IFN genes [64]. Studies have shown that VACV can interfere with signaling via both endosomal (TLRs 3, 8, and 9) and plasma membrane-bound (TLRs 2 and 4) receptors.

TLR3, an endosomal sensor specific for viral dsRNA, is capable of inducing both IRF3 and NF-κB activation. VACV proteins A52 and A46 are known to effectively interfere with different branches of TLR3 signaling. A52 blocks TLR3-mediated NF-κB activation induced by polyinosinic:polycytidylic acid (poly(I:C)), a synthetic dsRNA analog, by interacting with downstream adapter proteins such as IL-1 receptor-associated kinase2 (IRAK2) and tumor necrosis factor receptor-associated factor 6 (TRAF6) [65]. A46, on the other hand, inhibits IRF3 activation by binding to the adapter TIR domain containing adaptor molecule 1 (TICAM1), thereby preventing it from transmitting signals from TLR3 [66]. Together, A52 and A46 effectively shut down both arms of the TLR3 signaling pathway.

Interestingly, in vivo studies have shown that TLR3-deficient mice display reduced morbidity following VACV infection and exhibit lower viral replication in the respiratory tract. This suggests that VACV may exploit functional TLR3 to its advantage.

Other endosomal TLRs, such as TLR8 and TLR9, recognize viral nucleic acids—single-stranded (ss)RNA and DNA, respectively. The VACV E3 protein has been shown to inhibit cytokines expression induced by TLR8 [67] and TLR9 ligands [68]. Notably, the DNA-binding domain of E3 alone is sufficient to block TLR9 signaling [68]. Since E3 also contains RNA-binding domain that inhibits dsRNA-sensing receptors, it likely serves as a broad-spectrum shield that protects both VACV RNA and DNA from antiviral detection mechanisms.

VACV can also interfere with signaling from cell membrane-bound TLRs, including TLR2 and TLR4, although it remains unclear whether the virus can be directly recognized by these receptors [59].

### 3.5. NF-κB, IFNs and Other Cytokines

In addition to inhibiting foreign nuclear acid-recognition systems, VACV can also downregulate NF-κB signaling, a critical pathway for inducing many pro-inflammatory cytokines essential for combating viral infections. The N1 protein targets the IκB kinase (IKK) complex, inhibits NF-κB signaling via the TNF superfamily of receptors, and blocks NF-κB activation through Toll-like receptors [69]. The F14 protein binds to the transcriptional co-activator CREB-binding protein (CBP) and disrupts its ability to acetylate the p65 subunit of NF-κB, thereby impairing NF-κB’s capacity to recruit the transcription regulator bromodomain-containing protein 4 (BRD4). This leads to reduced expression of chemokine genes such as CCL2 and CXCL10 [70].

Another protein, B14, binds to the dimerization and kinase domain of IKKβ, preventing it from phosphorylating IκBα [40]. Additionally, the A49 protein mimics IκBα and is phosphorylated by IKKβ, but it then blocks the E3 ubiquitin ligase responsible for IκBα degradation [71]. Both mechanisms result in the accumulation of unphosphorylated IκBα, which sequesters NF-κB in the cytoplasm and prevents its activation and nuclear translocation. Furthermore, two other VACV proteins, K1 and A55, also contribute to NF-κB suppression, either by interfering with its activation or by blocking the translocation of the NF-κB heterodimer [39].

Another strategy employed by VACV to subvert host immune responses is the use of soluble cytokine decoys that inhibit host cytokine signaling [72]. The B18 protein, coded by the B18R gene, acts as a broad-spectrum decoy receptor for type I IFNs (IFN-α and IFN-β). Secreted from infected cells, B18 binds IFNs from a wide range of species, preventing their interaction with cell surface receptors. B18 can also attach to the external membranes of both infected and uninfected neighboring cells via glycosaminoglycans, creating a barrier that intercepts interferons before they can engage their receptor [73].

Even if IFN receptors are activated, VACV can still block signaling through the viral phosphatase H1, which dephosphorylates the STAT1 transcription factor, thereby preventing its nuclear translocation and inhibiting the expression of IFN-stimulated genes [74].

In addition, VACV produces a pair of secreted proteins known as cytokine response modifiers (CrmC and CrmE) that target soluble TNF-α. These proteins possess an N-terminal domain homologous to TNFRs, enabling them to sequester TNF-α from the extracellular milieu. While CrmC functions primarily as a free soluble molecule, CrmE can also associate with the cell membrane, blocking TNF-α from binding its receptor in a manner similar to B18’s inhibition of IFNs [75].

Other VACV-encoded cytokine decoy proteins include B8, which neutralizes IFN-γ [76]; viral IL-18-binding protein (vIL-18BP), which blocks IL-18 [77], and B15, which captures IL-1β [78]. Collectively, these immune evasion strategies allow VACV to evade early detection by the innate immune system and delay the onset of the adaptive response, that finally is responsible for clearing the infection.

### 3.6. Complement Evasion

The complement system is a network of cell-associated and soluble proteins that react with the surface of pathogens, leading to their lysis and inactivation [79]. Complement activation can be initiated through three pathways: the classical pathway, triggered by immunoglobulin-bound pathogens; the lectin pathway, activated by mannose-binding lectin (MBL); and the alternative pathway, which involves direct recognition by complement components. This activation is a multistep cascade process that culminates in the formation of membrane attack complex (MAC), a structure composed of enzymes that effectively lyse pathogens such as viruses, bacteria, and parasites [79].

To evade complement-mediated destruction, VACV has evolved a protein called VCP (VACV complement control protein), encoded by the gene C21L. VCP interferes with key complement components C3b and C4b, which are crucial for the function of C3 convertase—the central complex responsible for amplifying the complement signal, and the formation of C5 convertase, which drives MAC assembly. Additionally, C3b acts as an opsonin, coating viral particles to target them for phagocytosis.

Since all three complement pathways converge at the stages of C3 and C5 convertases, VACV can efficiently disrupt the entire system with a single protein. VCP also serves as a cofactor for the serine protease factor I, promoting the cleavage and inactivation of C3b and C4b and accelerating the decay of the C3 convertase complex [80]. By neutralizing complement activity, VACV effectively modulates both innate and adaptive immune responses.

### 3.7. Antigen Processing and Presentation

The adaptive immune response, driven by the activity of T and B lymphocytes, depends on the precise recognition of specific antigens associated with pathogens. To evade this response, VACV has evolved mechanisms to interfere with the processing and presentation of its antigens to the immune system.

Studies have shown that VACV infection impairs the ability of antigen-presenting cells (APCs) to display peptide antigens in the context of Major Histocompatibility Complex class II (MHC II) molecules. This effect is observed in infections with both replication-competent and replication-defective forms of the virus. Interestingly, the expression and cell surface levels of MHC II molecules remained unchanged, suggesting that VACV disrupts antigen loading rather than MHC II expression [81].

Further research identified the VACV protein A35, encoded by the A35R gene, as a key factor in this immune evasion. A35 localizes to endosomes and reduces the amount of peptide presented on MHC II molecules [82]. Its expression inhibits the activation of CD4^+^ T lymphocytes by APCs and diminishes their production of nitric oxide (NO) and cytokines in response to antigen stimulation. Although A35 is not essential for viral replication in vitro, its absence significantly attenuates viral virulence in vivo, underscoring the critical role of CD4^+^ T cell–mediated responses in viral clearance [82].

## 4. Strategies to Avoid Cell Death Mechanisms

**Figure 4:**
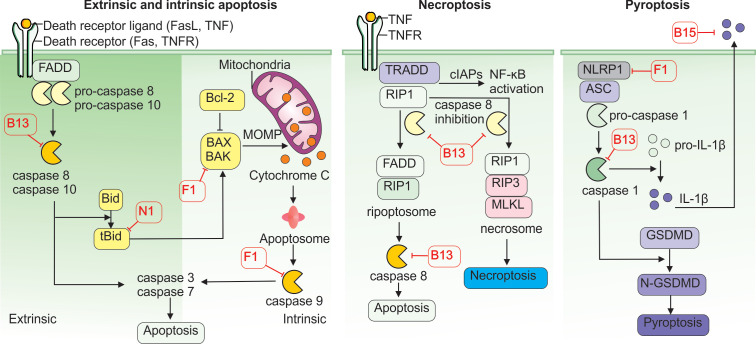
Inhibition of cell death pathways by vaccinia virus proteins. VACV employs multiple strategies to inhibit host cell death pathways and promote viral replication. The VACV F1 disrupts the intrinsic apoptosis pathway by blocking caspase-9 activation and suppressing pro-apoptotic factors BAX and BAK. Additionally, B13 functions as a pan-caspase inhibitor, blocking the death receptor-mediated extrinsic apoptosis pathway, whereas N1 blocks this signaling cascade by targeting the pro-apoptotic protein tBid. B13 also inhibits caspase-8 activation, and in its inactive form, caspase-8 allows the stabilization of RIP1 and RIP3, which interact via the RIP homotypic interaction motif (RHIM). This complex promotes the phosphorylation and oligomerization of MLKL (mixed lineage kinase domain-like protein), ultimately leading to plasma membrane rupture and necrotic cell death. VACV can also counteract pyroptosis using F1, which blocks the NLRP1 inflammasome. In addition, B13 inhibits caspase-1 intracellularly, and B15 acts extracellularly as a soluble IL-1β receptor.

Programmed cell death is a fundamental defense mechanism that helps prevent viral spread within the host. If an infected cell undergoes death before the virus can complete replication, it fails to produce progeny virions and is eliminated through phagocytosis by immune cells such as dendritic cells and macrophages [83,84]. Consequently, VACV evolved multiple tools to control mechanisms of programmed cell death and utilize them to its advantage (Figure 4).

Apoptosis is the most common form of programmed cell death. It can be triggered by internal or external signals and relies on caspases—a family of cysteine proteases that cleave target proteins at aspartic acid residues. Two major apoptotic pathways exist: extrinsic and intrinsic.

The extrinsic pathway is initiated by the activation of specific cell surface receptors, such as the TNFR and the Fas-ligand (FASL) receptor. Activated receptors induce the formation of a protein complex composed of death receptor adaptor proteins—TNFR-associated death domain (TRADD) and/or Fas-associated death domain (FADD)—which in turn activate the intracellular protease caspase-8. The activated caspase-8 then triggers downstream caspases-3 and -7, which cleave several cellular substrates, such as nuclear lamins and gelsolin, leading to DNA fragmentation and cell death [83].

The intrinsic pathway is activated by cellular stress such as DNA damage, oxidative stress, or nutrient deprivation, resulting in the permeabilization of the outer mitochondrial membrane. This process is mediated by Bax and Bak proteins, which form pores in the membrane, allowing cytochrome c to escape into the cytosol. Cytochrome c binds to apoptotic protease-activating factor-1 (APAF-1), forming a complex that activates caspase-9. Caspase-9, in turn, activates caspases-3 and -7, culminating in apoptosis [83].

Both apoptotic pathways can be triggered during viral infection, and VACV has evolved mechanisms to counteract them. The VACV protein B13 binds and inhibits both caspase-8 and caspase-9, effectively blocking the extrinsic and intrinsic pathways simultaneously [85]. Another viral protein, F1, inhibits both the precursor and active forms of caspase-9 and prevents the activation of caspases-3 and -7. F1 also interferes with Bak’s ability to bind Bax and form pores in the mitochondrial membrane. In addition, F1 blocks the pro-apoptotic protein Bim, a key antagonist of the anti-apoptotic factor Bcl-2. These actions are reinforced by the N1 protein, which neutralizes other Bcl-2 antagonists, Bid and Bad [86]. As a result, the mitochondrial pathway of apoptosis is effectively dismantled in VACV-infected cells. Other VACV proteins, such as B22, Golgi anti-apoptotic protein (GAAP), and E3, have also been implicated in blocking various stages of apoptosis [85].

In addition to apoptosis, VACV can inhibit pyroptosis, another form of programmed cell death that is dependent on the activation of caspase-1 via the inflammasome—a multiprotein complex. Pyroptosis is a highly pro-inflammatory process, typically triggered when intracellular PRRs such as NOD-like receptors (NLRs), especially NLRP1 (nucleotide-binding oligomerization domain, leucine-rich repeat and pyrin domain-containing 1), detect PAMPs. This recognition initiates the assembly of the adaptor protein ASC (apoptosis-associated, speck-like protein containing a CARD), which activates caspase-1. Activated caspase-1 processes pro-interleukin-1β (pro-IL-1β) into its active form and also activates gasdermin D (GSDMD), which forms pores in the cellular membrane, releasing pro-inflammatory contents [87].

Although commonly associated with bacterial infections, pyroptosis also plays a role in VACV infection [88]. VACV’s F1 protein binds the NLRP1 subunit, preventing inflammasome assembly and caspase-1 activation [88]. Additionally, B13 can directly inhibit caspase-1 activity [85], while B15 acts as a soluble decoy receptor for IL-1β, neutralizing pyroptosis-induced inflammatory signals and suppressing immune cell activation [78].

Interestingly, when apoptosis is inhibited in VACV-infected cells, necroptosis, another form of programmed cell death, may be triggered. Necroptosis occurs when caspase-8 is inhibited and cannot cleave two of its downstream targets, receptor-interacting serine/threonine kinase 1 (RIP1) and its relative RIP3. In this context, RIP1 and RIP3, along with inactive caspase-8, form the ripoptosome complex, which activates mixed lineage kinase domain-like pseudokinase (MLKL), a protein that oligomerizes and forms pores in the cell membrane, leading to cell lysis and the release of intracellular contents [89]. Because B13 strongly inhibits caspase-8, it can induce inadvertently promote ripoptosome formation and necroptosis [90].

This strategy offers dual benefits to VACV: by delaying apoptosis, VACV gains time to complete replication and produce progeny virions; and by inducing necroptosis, it facilitates viral release and dissemination to neighboring cells.

## 5. Interactions with Immune Cells

### 5.1. Dendritic Cells

As a pathogen, VACV interacts with various immune cell types, either directly by infecting them or indirectly by altering immune system function. Studies have shown that VACV exhibits a preference for infecting myeloid cells such as monocytes and dendritic cells (DCs), rather than B or T lymphocytes, although both lymphocyte types can still be infected [91].

Dendritic cells play a central role in the immune response by presenting viral antigens and initiating T cell-mediated immunity. In one study, VACV infection of immature DCs derived from peripheral blood mononuclear cells (PBMCs) impaired their maturation, as evidenced by reduced expression of key maturation markers including CD83, CD86, Human Leukocyte Antigen–DR isotype, (HLA-DR), and CD25 [92]. These infected DCs also showed a diminished capacity to stimulate T cell proliferation and exhibited increased levels of apoptosis. Notably, immature DCs were found to be significantly more susceptible to VACV infection than their mature counterparts [92].

Similar findings were observed in murine models: bone marrow-derived DCs failed to mature following VACV infection, as indicated by reduced expression of CD40, CD80, and CD86, diminished production of pro-inflammatory cytokines, and impaired activation of CD8^+^ T lymphocytes [93]. In contrast, mature DCs displayed greater resistance to VACV infection, with a lower proportion of infected cells and preserved antigen presentation and CD8^+^ T cell activation capabilities [93].

Interestingly, studies in mice infected with VACV reported that viral infection may actually promote DC maturation. Ex vivo analysis of splenic dendritic cells from VAVCV-infected mice showed increased expression of MHC I and co-stimulatory molecules CD40 and CD86 on their cell surface [94]. These DCs also exhibited elevated levels of IFN-β and demonstrated enhanced capacity to produce IL-10 and IL-12 upon LPS stimulation [94]. While these findings appear to contradict earlier results, they may be reconciled by the observation that fewer than 1% of splenic DCs were directly infected by VACV. This suggests that increased maturation may have occurred in uninfected DCs in response to cytokine signaling, particularly type I IFNs.

Conversely, the same study found that VACV infection reduced expression of MHCII on DCs and impaired their ability to present antigens to CD4^+^ T lymphocytes [94], a disruption potentially attributable to the viral A35 protein. Collectively, these findings suggest that DCs respond to VACV infection by preferentially activating CD8^+^ cell responses over CD4^+^ T cell responses, which aligns with the immune system’s typical strategy for combating viral infections.

### 5.2. Macrophages

Macrophages are another type of immune cell that can be infected by VACV. Studies have demonstrated that VACV is capable of infecting and replicating in both human M1 and M2 macrophages in vitro, with the M2 subset producing more than twice the viral titer per cell compared to M1 macrophages [95]. Infected macrophages exhibited cytoplasmic viral factories, while levels of apoptosis and necrosis remained low even 48 h post-infection. Notably, activation of macrophages with LPS and IFN-γ did not affect viral replication, whereas stimulation with IL-10 or a combination of LPS and IL-1β significantly inhibited viral production.

Interestingly, the majority of virions generated in macrophages were EEVs, which are primarily involved in long-range viral dissemination. Given the migratory capacity of macrophages between tissues, these findings suggest that macrophages may play a critical role in the systemic spread of the virus within the host [95]. In contrast, monocytes examined in the same study supported only minimal VACV replication and exhibited high levels of apoptosis, indicating a less permissive environment for viral propagation [95].

### 5.3. Neutrophils and NK Cells

While the role of neutrophils in VACV infection has not been extensively characterized, studies suggest they contribute significantly to early virus clearance and the initiation of adaptive T cell responses. Experiments using oncolytic VACV demonstrated that neutrophils internalize more virus than any other cell subset, accounting for over 80% of viral uptake and subsequently degrade the virus within phagocytic vesicles [96]. Interestingly, depletion of neutrophils using anti-Ly6G antibodies significantly enhanced the efficacy of oncolytic VACV in treating B16F10 tumors [96]. Additionally, neutrophils have been shown to transport VACV antigens to the bone marrow, where they deliver them to antigen-presenting cells, thereby promoting the development of CD8^+^ T cell responses [97].

Natural killer (NK) cells also play an important role in the immune response to VACV. Although VACV can directly infect NK cells, the functional consequences of this infection remain poorly understood [96]. Nonetheless, studies have shown that NK cells are recruited to sites of VACV infection, where they become activated, proliferate, and lyse infected cells [98,99,100]. Notably, VACV infection does not reduce MHC I expression or the expression of ligands for the NK Group 2 Member D (NKG2D) receptor on infected cells. Instead, it induces the expression of ligands for other activating NK cell receptors, including NKp46, NKp44, and NKp30 [100]. In a mouse model of pulmonary VACV infection, NK cells were found to produce high levels of IFN-γ prior to the infiltration of CD8^+^ T cells, helping to limit viral replication during the early stages of infection [98]. Furthermore, NK cells were observed to form a memory-like Thy+ subset capable of protecting naïve immunodeficient mice from lethal VACV challenge [101].

### 5.4. B and T Lymphocytes

VACV also interacts with components of the adaptive immune system. B cells, which produce virus-neutralizing antibodies, can also function as APCs to stimulate T cell responses. Although VACV can bind to multiple B cell subpopulations, it primarily infects and replicates in memory B cells [102]. However, successful viral replication requires B cell activation; in the absence of stimulation, infection is abortive and late gene expression is not induced [102].

A murine model of respiratory VACV infection revealed that virus-specific antibodies begin to appear around day 15 post-infection, peak at day 148, and play only a minor role in controlling primary infection [103]. In contrast, serum from mice with a fully developed B cell response was highly effective in mitigating the effects of subsequent VACV exposure [103]. Although the B cell response to VACV develops slowly, it results in durable immune memory. In humans, memory B cells and protective antibodies specific to the VACV-based smallpox vaccine have been detected up to 50 and 59 years post-vaccination, respectively [104]. Similarly, in mice, VACV induces a robust B cell memory response, though its protective role appears secondary to that of memory T cells [105].

T cells, while only weakly susceptible to VACV infection [106], play a central role in viral clearance. Experimental data show that CD8^+^ T lymphocytes are both necessary and sufficient to protect mice against VACV respiratory infection [103]. Depletion of CD8^+^ cells renders immunocompetent mice susceptible to VACV-induced mortality, whereas adoptive transfer of naïve CD8^+^ T cells rescues immunodeficient RAG-/- mice from lethal infection [103]. Although CD4^+^ T cells are essential for the development of a functional antibody response, their depletion does not impair survival or virus clearance in the respiratory model [103]. Interestingly, in an intraperitoneal model of VACV infection, CD4^+^ T cells played a more substantial role by supporting proper CD8^+^ T cell priming [107].

Additional evidence points to a role for γδ T cells in CD8^+^ T cell activation. These cells can present VACV antigens via MHC I and secrete cytokines such as IL-1 and IFN-α, thereby contributing to antiviral defense [108].

Like B cells, T cells also generate robust and long-lasting memory. In humans, VACV-specific CD8^+^ memory T cells have been detected up to 50 years post-vaccination, maintaining cytotoxic activity against infected cells [109]. Remarkably, CD4^+^ memory T cells isolated form these individuals also exhibited cytotoxic, rather than regulatory, properties [109].

## 6. VACV as a Tool for Oncolytic Cancer Therapy

### 6.1. Advantages of VACV as an Anti-Cancer Agent

VACV possesses several characteristics that make it an exceptionally valuable tool for oncolytic cancer therapy. First, it can infect a wide range of cell types. While many viruses, such as adenoviruses, are restricted to infecting cells that express a specific receptor, VACV can infect tumors originating from various tissues [1,19,110,111]. This gives it advantage over, for instance, Herpes Simplex Virus 1 (HSV-1), which is also used as oncolytic vector but displays preferential neurotropism and, in the case of non-neural cancers, can only be administered intratumorally [112]. Moreover, VACV binds to MARCO, a protein commonly found on tumor-associated macrophages and myeloid-derived suppressor cells (MDSCs). This property makes it a promising candidate for modifying the tumor microenvironment, for example, by delivering genes that reverse the immunosuppressive phenotype of myeloid cells.

The ability of VACV to attach to a variety of cell types is particularly advantageous because tumors, especially at advanced stages, are highly heterogenous, with multiple subpopulations of cells expressing different receptors and surface markers. As a result, oncolytic agents that rely on a single receptor type have limited capacity to infect diverse tumor cells and produce significant therapeutic effects. In contrast, VACV can enter multiple types of tumor cells and destroy them without depending on a specific receptor. Therefore, as long as VACV replication remains confined to cancer cells and does not significantly affect healthy tissues, its broad, receptor-dependent tropism is not a limitation but rather a benefit.

Second, the large genome of VACV allows for the insertion of sizable therapeutic transgene constructs of at least 25 kb [113,114]. This gives VACV an advantage over other types of vectors, such as adenoviruses, which, in order to remain replication-competent, have a more limited capacity, typical of constructs smaller than 10 kb [115]. Additionally, because VACV replicates entirely in the cytosol, there is no risk of transgene integration into the host’s genome, thereby minimizing the chance of transformation if normal cells are infected.

Third, VACV has a rapid replication cycle. It produces new virions within 6–8 h and causes lysis of infected cells within 48 h, enabling it to quickly infect and destroy tumor cells before the immune system can neutralize the virus. Notably, VACV replicates at comparable rates under both normoxic and hypoxic conditions, making it particularly effective against tumors in low oxygen environments such as pancreatic cancer [116].

Additionally, VACV suppresses proteins involved in apoptosis, allowing it to replicate efficiently in cancer cells with defective apoptosis pathways. While resistance to apoptosis undermines many conventional therapies, it actually enhances VACV replication by prolonging host cell survival. Instead of apoptosis, VACV induces necroptosis and cell lysis, resulting in the release of damage-associated molecular pattern (DAMP) molecules such as high-mobility group box 1 protein (HMGB1), adenosine triphosphate (ATP), calreticulin (CRT), and heat shock protein 90 (HSP90) [117]. The release of these DAMPs characterizes immunogenic cell death (ICD), which promotes leukocyte recruitment, dendritic cell maturation, and priming CD8^+^ T lymphocytes against tumor antigens [118].

Consequently, VACV is especially promising for treating “cold” tumors—those with poor immune cell infiltration—by converting them into “hot” tumors actively infiltrated by NK and T cells. Finally, VACV induces a predominantly CD8^+^ cytotoxic T cell response, which is particularly beneficial in cancer therapy, as CD4^+^ T cells and B cells can sometimes exert immunosuppressive effects.

### 6.2. Chimeric VACV: CF33

**Figure 5:**
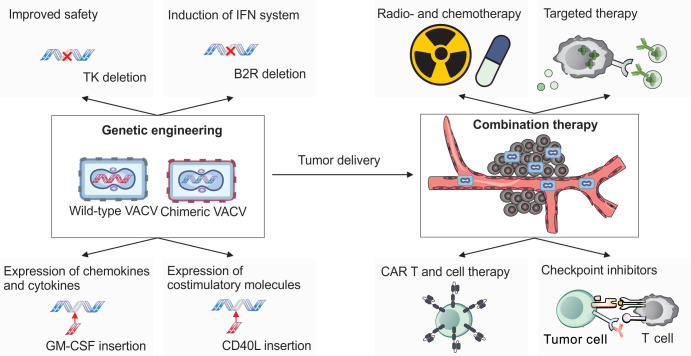
Vaccinia virus in cancer therapy. Wild-type or chimeric strains of VACV can be genetically engineered in multiple ways to improve both safety and therapeutic efficacy for cancer treatment. Deletion of the thymidine kinase (TK) gene restricts viral replication to rapidly dividing tumor cells, improving tumor selectivity. Removal of the B2R gene enhances host interferon responses, further supporting anti-tumor immunity. Therapeutic transgenes can be inserted into the VACV genome to modulate the tumor microenvironment. For example, expression of cytokines and chemokines such as GM-CSF (granulocyte–macrophage colony-stimulating factor) or CCL5 (RANTES) promotes the recruitment and activation of immune cells within tumors. Additionally, the expression of co-stimulatory molecules such as CD40L or OX40L enhances T cell activation and survival. As an oncolytic agent, VACV can be employed either as a monotherapy or in combination with other treatments to improve therapeutic outcomes. Combination strategies include conventional approaches such as radio- and chemotherapy, as well as emerging modalities such as CAR-T cells and other adoptive cell therapies, immune checkpoint inhibitors, and targeted therapies involving antibody–drug conjugates and small-molecule inhibitors.

To further enhance the oncolytic properties of VACV, researchers have employed several strategies that are summarized in Figure 5. One such approach is chimerization, which involves combining genetic elements from different, closely related poxviruses to generate chimeric viruses with optimal oncolytic potential. In a study conducted at City of Hope, researchers co-infected cells with cowpox, raccoonpox, rabbitpox, and six different strains of VACV to generate a diverse pool of chimeric orthopoxviruses. From this pool, 100 chimeric viruses were isolated and screened using high-throughput methods for their efficacy against the NCI-60 panel of human cancer cell lines [119,120]. However, it should be noted that 2D cell cultures do not capture the complexity of real tumors, including tumor microenvironment interactions, the effects of the host immune system, and the heterogeneity of tumor architecture. As a result, findings from 2D cultures may overestimate therapeutic efficacy while underestimating potential toxicity or immune interactions.

Among these candidates, the chimeric virus CF33 demonstrated superior oncolytic activity compared to its parental strains, including robust cytotoxicity against six different human pancreatic cancer cell lines. It also exhibited a strong ability to induce immunogenic cell death and significantly inhibited the growth of pancreatic and triple-negative breast cancer xenografts in mouse models. Interestingly, although CF33 was generated through gain-of-function chimera screening and lacks deletions of viral virulent genes such as TK, it nonetheless showed impressive safety profiles in animal models [119,120]. Sequence analysis of the CF33 genome revealed that it is primarily derived from three VACV strains—IHD, Lister, and WR—which together account for approximately 60% of its genome. No genetic material from raccoonpox or cowpox viruses was detected, indicating that CF33 is in fact a chimeric VACV, rather than a broader orthopoxvirus hybrid [121].

Building on CF33, several derivative viruses have been developed. One such derivative, CF33-hNIS (also known as VAXINIA), incorporates the human sodium iodide symporter (hNIS) gene, enabling non-invasive imaging of viral distribution via using PET scans. This modification allows researchers to track the virus’s localization and replication within the body [122].

Another derivative, CF33-hNIS-antiPDL1, expresses both hNIS and a single-chain variable fragment targeting PD-L1, an immune checkpoint protein. Preclinical studies in models of triple-negative breast cancer and gastric cancer peritoneal metastases have shown that this dual-function virus not only enhances direct tumor cell killing but also reprograms the tumor microenvironment to improve immune recognition and activation [121,123].

A third variant, CF33-CD19, is engineered to express a truncated CD19 on the surface of infected cancer cells. This strategy enables targeting of solid tumors with CD19-specific CAR-T cells, which typically show limited efficacy against solid tumors due to the lack of appropriate surface antigens [124].

All three CF33-based viruses are currently under clinical evaluation:

CF33-hNIS is being tested as a monotherapy or in combination with pembrolizumab in adults with metastatic or advanced solid tumors (NCT05346484),

CF33-hNIS-antiPDL1 is in clinical trials for the treatment of metastatic triple-negative breast cancer (NCT05081492),

CF33-CD19 is being evaluated as a monotherapy or in combination with blinatumomab for metastatic solid tumors (NCT06063317).

### 6.3. Genetic Engineering of VACV

Creating novel oncolytic variants of VACV typically involves precise insertions and deletions within the viral genome. However, due to its large genome size (~195 kb), VACV is not amenable to standard molecular cloning techniques commonly used for smaller viral vectors, such as lentiviral systems. Consequently, genetic engineering approaches are required.

The most traditional and widely used method is based on homologous recombination. In this approach, a plasmid carrying the desired genetic construct is introduced into VACV-infected cells. Recombination occurs between homologous sequences on the plasmid and the viral genome, enabling the exchange of genetic material [125]. A typical construct includes a desired transgene (e.g., a cytokine) accompanied by a selection marker (e.g., GFP or β-galactosidase), each driven by separate promoters. The expression cassette is flanked by sequences homologous to regions of the viral genome—commonly the termini of the TK gene—to allow for targeted insertion. Recombinant viruses are isolated using marker-based selection or screening, followed by multiple rounds of plaque purification.

While conceptually straightforward and technically accessible, this method is labor-intensive and inefficient: recombination events occur at low frequency, yielding approximately one recombinant per 1000 wild-type virions [9].

To enhance efficiency, several alternative strategies have been developed, including the use of bacterial artificial chromosomes (BACs) [126] and genome-editing techniques based on CRISPR-Cas9 [127]. A method, known as MAVERICC (marker-free vaccinia virus engineering of recombinants through in vitro CRISPR/Cas9 cleavage) integrates the strengths of previous approaches [9].

In the MAVERICC method, the VACV genome is first cleaved in vitro at specific sites using Cas9 guided by sequence-specific RNAs. This cleaved genome is then co-transfected with an amplicon containing the desired transgene flanked by sequences homologous to the targeted genomic region. Recombination occurs within the host cell, which is also infected with a replication-defective helper poxvirus. The helper virus supplies the necessary proteins to facilitate homologous recombination but cannot replicate itself. As a result, only recombinant VACV that have repaired the Cas9-induced cleavage through homologous recombination with the amplicon can be propagated. This method enables the generation of engineered viruses with >90% efficiency, without the need for selection markers, serial passaging, or extensive screening [9].

Alternative approaches involve the use of an antibiotic resistance gene inserted into the construct that recombines with the viral genome. In this strategy, after recombination, viruses are seeded onto cells treated with an antibiotic that blocks protein synthesis. As a result, only viruses that have successfully recombined and contain the antibiotic resistance gene can carry out protein synthesis and produce a new generation of virions.

The latest iteration of this method combines antibiotic selection with the use of transposons and long-read nanopore sequencing. In this approach, transposons containing both the antibiotic resistance gene and the desired construct are transfected into cells infected with VACV. After selection, viral genomes are analyzed using long-read nanopore sequencing, which enables precise characterization of the introduced mutations. With this strategy, the desired modification of VACV can be achieved in as quickly as 5 days [128].

### 6.4. Commonly Targeted Viral Genes for Modification in VACV-Based Oncolytic Viruses

Over the years, numerous strategies have been developed to enhance the suitability of VACV for cancer therapy [1]. Cancer cells possess several characteristics that facilitate enhanced replication of VACV, ultimately leading to cell lysis. These include deficient apoptotic pathways [129], reduced expression of tumor suppressor genes (such as p53), and impaired antiviral and interferon signaling mechanisms [130]. In addition, due to their high proliferative rates and intense DNA synthesis demands, cancer cells typically exhibit elevated levels of cytosolic TK and RR [131,132].

To exploit this metabolic environment, researchers have engineered VACV strains with deletions in the viral genes encoding TK and/or RR. These modified viruses rely on host cell-derived enzymes to synthesize DNA precursors, thereby restricting viral replication to cancer cells where these enzymes are abundant [133,134]. Interestingly, endothelial cells in tumor-associated vasculature also exhibit elevated TK expression, largely due to stimulation by vascular endothelial growth factor (VEGF). This makes VACV a potential anti-angiogenic agent. For example, the oncolytic strain JX-594—engineered to express human granulocyte–monocyte colony-stimulating factor (hGM-CSF) and β-galactosidase (β-gal)—has been shown to selectively replicate within tumor-associated blood vessels, causing their collapse while sparing normal vasculature [135].

Another modification involves deletion of the C11R, which encodes the viral growth factor (VGF). VGF stimulates the EGFR-Ras signaling pathway to promote cell proliferation. In the absence of VGF, VACV replication is impaired unless the host cell has an active EGFR pathway—a common feature of many cancer cells [136].

Additional deletions used to enhance oncolytic specificity and safety include A56R and F14.5L. The A56R gene encodes a hemagglutinin-like protein (A56) that prevents infected cells from forming syncytia, protects against superinfection, and inhibits complement-mediated lysis [137]. Despite these roles, A56 is not essential for viral replication and is often replaced with therapeutic transgenes. The F14.5L gene is involved in regulating cell adhesion and contributes to viral virulence in vivo, though it is not required for replication [138]. Its deletion is commonly employed as a safety measure, especially important for treating potentially immunocompromised cancer patients [139]. Other deletions introduced to increase safety and tumor specificity include genes such as SPI-1 (B22) and -2 (B13), B18R, N1L, A41L, A49L, and F1L. A list of these deletions and their associated viruses is provided in Table 1.

**Table 1:** Examples of viral genes deleted from vaccinia virus to enhance tumor selectivity and promote immune system activation.

Deleted Viral Genes	Functions of Deleted Viral Genes	Example of Virus	References
TK (Thymidine kinase)	Provides material for viral DNA replication	vCB2 (vvLuc)	[134]
TK and VGF (Viral growth factor)	Supports viral replication, accelerates cell growth and metabolism	vvDD-GFP	[140]
SPI-1 (B22) and -2 (B13)	Inhibit apoptosis	vSP	[141]
TK, SPI-1 and -2	Supports viral replication, inhibit apoptosis	vSPT	[142]
Soluble type I IFN receptor (B18R)	Inhibits mobilization of the immune cells	WR-delB18	[143]
Soluble type I IFN receptor and TK	Inhibits mobilization of the immune cell, supports viral replication	ΔB18RΔTK	[143]
F14.5L, TK, and HA	Promotes virulence in vivo, supports viral replication, inhibits complement-mediated lysis of infected cell	GLV-1h68	[144]
TK, RR (ribonucleotide reductase)	Support viral replication by providing material for DNA synthesis	TG6002	[145]
TK, N1L, and A41L	Supports viral replication, inhibits apoptosis, interferes with chemokine signaling	VVLΔTKΔN1LΔA41L	[146]
A49L	Inhibits NF-κB activation	vΔA49L	[147]
TK, F1L	Supports viral replication, inhibits apoptosis	ΔTK/F1L	[148]
TK, B2R	Supports viral replication, inhibits the cGAS/STING pathway	WR/TK−/ΔB2	[149]

### 6.5. Expression of Chemokines and Cytokines

Many VACV-based oncolytic viruses are engineered to express immunostimulatory genes, primarily chemokines and cytokines, to enhance anti-tumor immunity. These modifications generally pursue two main objectives: (1) to recruit antigen-presenting cells, particularly DCs, promote their maturation, and improve their capacity to present antigens to T cells; (2) to modulate existing T cell responses by promoting T cell survival, sustaining activation, and preventing exhaustion.

One of the most commonly used transgenes is GM-CSF, which is known to promote tumor infiltration by NK and DCs and accelerates DC maturation [150]. GM-CSF has been used as an immunostimulant in a variety of oncolytic viruses, including adenovirus [151], Sindbis [152], and the only FDA-approved oncolytic virus in the U.S., the HSV-based T-VEC [153]. Multiple VACV vectors engineered to express GM-CSF have shown promising results in preclinical studies [135,154,155].

An alternative approach strategy involves incorporating genes encoding chemokines such as CXCL11 and CCL5 (RANTES). A VACV strain expressing CCL5 induced significant infiltration of DCs, CD4^+^ T cells, and NK cells, effectively suppressing tumor growth in the MC38 murine model [156]. Similarly, another VACV expressing CXCL11 was highly effective in the same model, attracting large numbers of CD8^+^ lymphocytes and inducing strong IFN-γ expression [157].

To further enhance T cell-mediated responses, pro-inflammatory cytokine genes such as IL-12 [158], IL-15 [159], and IL-23 [160] have been inserted into the VACV genome. These modifications led to improved anti-tumor effects with increased CD8^+^ T cell infiltration, consistent with the roles of these cytokines in supporting cytotoxic T lymphocyte (CTL) proliferation, survival, and effector function.

Interestingly, the anti-inflammatory cytokine IL-10 also enhanced anti-tumor effects when expressed by oncolytic VACV in a mouse model of pancreatic cancer [161]. This finding is counterintuitive, given that IL-10 is generally associated with the suppression of immune responses, including inhibition of IL-12 production. The study revealed that IL-10 expressing VACV persisted longer within tumors, led to fewer VACV-specific CD8^+^ T cells, and resulted in reduced infiltration by macrophages [161]. This suggests that IL-10 may prolong viral replication within tumor tissue, thereby enhancing direct oncolysis and potentially facilitating a more robust anti-tumor immune response. Additionally, IL10 is known to impair macrophage antigen presentation to CD4^+^ cells [162], potentially skewing the immune response toward a CD8^+^ T cell-driven cytotoxic pathway.

### 6.6. Induction of the IFN System

Robust activation of the type I IFN response is critical for the development of effective cytotoxic immunity, which is a key objective in cancer immunotherapy [163]. However, VACV encodes multiple genes that inhibit the host IFN response, posing a challenge for its use as an oncolytic agent. To address this, researchers have developed strategies to prevent VACV from suppressing IFN production. One such strategy involves deletion of the B2R gene, which encodes a nuclease that degrades cGAMP—a key activator of the cGAS-STING pathway [149]. Another targeted gene is E5R, a gene that promotes degradation of cGAS itself [50]. VACV strains lacking B2R exhibit elevated IFN expression and enhanced anti-tumor activity in vivo [149]. Notably, B2R deletion also reduces viral virulence, making the modified virus potentially safer for use in immunocompromised patients.

An alternative strategy involves the insertion of the gene-encoding, DNA-dependent activator of IFN-regulatory factors (DAI), which can activate IRF3 through a cGAS-independent pathway [164]. In one study, DAI-expressing VACV demonstrated significantly improved inhibition of melanoma tumor growth in both syngeneic mouse models and humanized mice, even when the B2R gene remained intact [164].

A more novel approach utilized the gene-encoding, white-spotted charr lectin (WCL), a plant-derived protein previously linked to strong anti-tumor effects. VACV engineered to express WCL gene was shown to robustly activate IRF3 and induce high levels of type I IFNs, leading to the effective suppression of hepatocellular carcinoma in a mouse model [165].

### 6.7. Expression of Co-Stimulatory Molecules

To boost the activity of tumor-primed T cells, various research groups have developed VACVs encoding co-stimulatory molecules such as CD40L, 4-1BBL, and OX40L. Signals derived from these molecules are known to enhance T cell activity, reduce apoptosis, and increase the production of pro-inflammatory cytokines. When delivered intratumorally, these engineered viruses successfully inhibited the growth of B16 melanoma tumors in immunocompetent mice and extended the survival of the treated animals [166,167,168].

Additionally, a CD40L-expressing VACV effectively slowed the progression of bladder cancer in a mouse xenograft model by activating the NF-κB pathway in T cells and promoting the secretion of TNF-α, IL-1α, and RANTES [167]. In another study, a VACV encoding a tandem of Fms-related tyrosine kinase 3 ligand (Flt3l) and OX40L genes completely eliminated A20 lymphoma tumors and significantly delayed the development of spontaneous tumors in the MMTV-PyMT mouse model of triple-negative breast cancer (TNBC) [168]. The use of the OX40L/FLt3l-expressing virus also led to a marked depletion of regulatory T cells (Tregs), reprogramming them into a more cytotoxic-like phenotype [168].

### 6.8. Other Strategies

Researchers have also explored several innovative strategies to enhance the anti-tumor properties of VACV. In one notable study, a VACV engineered to express a secretory, bispecific T cell engager composed of single-chain variable antibody fragments specific for CD3 and the tumor antigen EphA. This virus effectively recruited T lymphocytes to EphA-expressing cancer cells and induced complete clearance of A549 tumors in SCID mice infused with human PBMCs [169].

Another approach targeted the transforming growth factor beta (TGF-β) pathway, which is frequently upregulated in immune-resistant tumors. A VACV expressing a soluble TGF-β inhibitor was able to eliminate head and neck squamous cell carcinoma (HNSCC) tumors that were resistant to treatment with a control VACV containing B2R and TK deletions. The engineered virus reduced the number of Tregs and increased their sensitivity to IFN-γ signaling [170].

Finally, to target tumor vasculature, scientists engineered a VACV to express the anti-VEGF single-chain antibody GLAF1. Treatment with this virus dramatically decreased blood vessel density within tumors and inhibited disease progression in xenograft models [171].

Overall, as our understanding of immune stimulation continues to grow, we can expect an increasing number of innovative genetic constructs to be tested as transgenes in the new generation of oncolytic VACVs.

## 7. Vaccinia Virus in Combination with Other Therapeutic Strategies

### 7.1. Combination with CAR-T Therapies

Although various forms of oncolytic VACV have demonstrated promising results as monotherapies in cancer treatment, many researchers are now exploring their use in combination with other therapeutic approaches. One notable strategy involves combining VACV with chimeric antigen receptor T cell (CAR-T) therapy.

CAR-T-based therapies are highly effective against CD19-expressing lymphomas; however, their efficacy in treating solid tumors remains limited due to the lack of tumor-specific antigens [172]. To overcome this challenge, researchers engineered VACV to express the CD19 gene. Because VACV preferentially replicates in tumor tissues, it can induce CD19 expression on the surface of cancer cells, rendering them susceptible to CAR-T cell-mediated cytotoxicity. This combination demonstrated promising results in the B16 melanoma model [173], as well as in the MC-38 colorectal cancer model and human tumor xenograft models [174].

In another approach, a CXCL11-expressing VACV was used to attract mesothelin-specific CAR-T cells toward mesothelin-positive TC-1 tumors. While both the virus and CAR-T cells individually inhibited tumor progression following intravenous injections, their combination produced a significantly more potent effect [175].

These findings suggest that VACV can serve as a valuable adjunct to enhance the efficacy of CAR-T therapies, particularly in the treatment of solid tumors.

### 7.2. Combination with Checkpoint Inhibitors

Since many oncolytic VACVs can activate immune responses in immunologically “cold” tumors, a logical strategy is to combine them with immune checkpoint inhibitors, which can sustain and amplify T cell activity once it has been initiated [176,177]. For example, the combination of IL-21-expressing VACV with an anti-PD-1 antibody produced a significantly stronger therapeutic effect in a mouse glioma model than either treatment alone [178]. Similar synergistic effects were observed in A20 and EL4 lymphoma models when combining a VACV expressing manganese superoxide dismutase (MnSOD) with anti-PD-L1 antibodies [179]. In the MC-38 colon cancer model, the efficacy of a CXCL11-expressing VACV was also substantially enhanced by co-administration of anti-PD-L1 therapy [180].

Because systemic administration of checkpoint inhibitors is often associated with immune-related toxicities, researchers have also explored engineering VACVs to express checkpoint-inhibitory molecules directly within tumors. Leveraging the virus’s tumor-specific replication, this approach allows for localized delivery of checkpoint inhibitors, potentially reducing systemic side effects. A VACV co-expressing a soluble PD-L1 inhibitor and GM-CSF demonstrated high efficacy in treating B16 melanoma tumors [181]. Another engineered virus encoding a cell-depleting anti-CTLA4 antibody and GM-CSF successfully eradicated tumors in multiple models, including breast (EMT6), colon (MC-38), and melanoma (B16) models, following intratumoral administration. Tumor regression in these models was associated with a marked reduction in CD4^+^ Treg cells and exhausted CD8^+^ T cells, alongside expansion of activated cytotoxic CD8^+^ T cells. Combining this therapy with anti-PD-1 antibodies further improved its therapeutic efficacy [182].

Another checkpoint target explored with VACV is TIGIT (T cell immunoreceptor with Ig and ITIM domains), a common marker of exhausted and regulatory T cells. Researchers engineered a VACV to express the variable domains of heavy and light chains of an anti-TIGIT antibody. This virus effectively slowed the progression of several subcutaneously implanted tumor models, including EMT6 (breast), CT26, MC-38 (colon), and H22 (liver) tumors. Once again, combining the treatment with anti-PD-1 therapy further enhanced the anti-tumor effects [183].

Collectively, these findings support the idea that immune checkpoint inhibitors, whether co-administered or encoded directly within the virus, are likely to play a critical role in the future development of VACV-based cancer therapies.

### 7.3. Combination with Radio- and Chemotherapy

Chemotherapy and radiotherapy remain among the most widely used cancer treatments, and the potential of combining these with VACV has been explored in several studies. Radiotherapy has been shown to enhance the efficacy of oncolytic VACV in preclinical models of glioblastoma [184,185] and pancreatic cancer [186]. In the TC-1 lung cancer model, combining VACV with radiotherapy increased tumor cell necroptosis and stimulated the release of DAMP molecules. This, in turn, activated T cells and reduced the populations of Tregs and M2 macrophages [187]. Owing to its tumor selectivity, VACV is a promising agent for sensitizing tumors to radiation.

One commonly employed strategy involved engineering VACV to express the sodium iodide symporter (NIS) [188,189,190]. Infection with this virus significantly increased the uptake of radioactive Iodine-131 (^131^I) in prostate cancer xenografts, leading to tumor growth inhibition and prolonged survival in treated mice [191]. In another strategy, researchers developed a radiotherapy system using VACV expressing the somatotropin receptor in combination with a radioisotope-labeled somatotropin analog. In both subcutaneous and disseminated mouse models of colorectal cancer, intraperitoneal administration of the virus resulted in tumor-specific localization and targeted uptake of the radiolabeled compound. This specific accumulation of radioisotopes within the tumor tissue significantly inhibited tumor growth, improved survival, and did not cause systemic toxicity [192].

VACV has also been studied in combination with conventional chemotherapy. Paclitaxel, for example, significantly enhanced the anti-tumor efficacy of VACV in HCT116 tumors grown in athymic mice. This effect was associated with type I IFN production and the release of HMGB1, a key DAMP molecule [193]. Furthermore, both cisplatin and gemcitabine improved the efficacy of oncolytic VACV in pancreatic tumor xenografts in nude mice [194]. Similarly, treatment with cyclophosphamide (CPA) or rapamycin increased VACV’s ability to inhibit the growth of malignant gliomas in rat models [195]. CPA also enhanced the efficacy of intravenously administered VACV in lung cancer xenografts by increasing viral spread within the tumor and reducing tumor vasculature [196].

Another commonly employed strategy involves arming VACV with genes that convert inactive prodrugs into active chemotherapeutic agents. Given VACV’s selectivity for tumor tissue, this approach allows high local concentrations of the active drug while minimizing systemic toxicity. For example, a VACV engineered to express super cytosine deaminase (SCD) effectively converted the prodrug 5-fluorocytosine (5-FC) into the chemotherapeutic compound 5-fluorouracil (5-FU). In the presence of prodrug, the virus induced death in cancer cell lines that were otherwise resistant to VACV-mediated lysis [197]. Another engineered VACV expressed β-galactosidase to activate a prodrug containing a β-galactosidase-specific cleavage site. In a breast cancer xenograft model, this virus–prodrug system led to accelerated tumor shrinkage and induced apoptosis in multiple cancer cell lines when the prodrug was present [198].

### 7.4. Combination with Small-Molecule Inhibitors

The oncolytic potential of VACV has also been evaluated in combination with various small-molecule inhibitors used in cancer therapy. Trametinib, a clinically approved inhibitor of mitogen-activated protein kinase MEK, was found to enhance VACV replication in vitro and improve its ability to inhibit the growth of ovarian cancer xenografts [199]. Idelalisib, an FDA-approved selective inhibitor of phosphoinositide 3-kinase delta (PI3Kδ), significantly increased viral delivery to tumors following intravenous injection and substantially enhanced the therapeutic effect [200]. Other notable inhibitors that have been shown to improve the anti-cancer activity of oncolytic VACVs include trichostatin A, a histone deacetylase inhibitor, and sunitinib, a multitargeted receptor tyrosine kinase inhibitor. Trichostatin A markedly increased viral replication and spread within tumor tissues [201], while sunitinib robustly enhanced CD8^+^ T cell infiltration, suppressed Tregs, and increased tumor cell apoptosis by more than threefold [202]. Inhibitors targeting the VEGF pathway have also been found to potentiate the oncolytic effects of VACV [171,203]. As the number of novel inhibitors targeting tumor-specific pathways continues to grow, combining them with VACV holds great promise for achieving even more effective cancer therapies.

## 8. Clinical Trials and Potential Obstacles

The specificity of VACV for cancer cells and the promising results from preclinical models have spurred the development of multiple VACV-based oncolytic viruses, many of which have progressed to clinical trials. A key consideration in patient treatment is the choice of viral delivery route. Systemic administration, such as intravenous or intraperitoneal injection, is the most convenient; however, it requires the virus to have high tumor-targeting specificity. Moreover, repeated systemic dosing can be challenging due to the development of neutralizing antibodies, which reduce the efficacy of subsequent treatments.

In contrast, intratumoral injection allows for direct delivery of the virus into the tumor, enabling the use of lower doses and minimizing the impact of circulating neutralizing antibodies. However, this method has its limitations—it is not feasible when tumors are inaccessible or numerous, and it often requires complex medical procedures. These procedures can be burdensome for patients and may affect their daily lives.

Current clinical trials involving VACV utilize both systemic and intratumoral delivery approaches. Table 2 summarizes clinical studies published between 2009 and 2025, while a comprehensive list of earlier clinical trials has been published elsewhere [204]. An up-to-date list of ongoing clinical trials involving VACV can be found at https://clinicaltrials.gov/(accessed on 3 July 2025). We also provide a summary of these ongoing trials in Table 3.

As of April 2025, only two trials involving oncolytic VACV have reached phase III. The first is a study evaluating intraperitoneal delivery of Genelux’s Olvi-Vec (GL-ONC1) in combination with a platinum-based chemotherapy regimen for the treatment of platinum-resistant refractory ovarian cancer (NCT05281471). Olvi-Vec was engineered by inserting three expression cassettes—encoding a *Renilla* luciferase–*Aequorea* green fluorescent protein fusion, β-galactosidase, and β-glucuronidase—into the F14.5L, J2R (encoding TK), and A56R (encoding hemagglutinin) loci of the parental VACV genome. In addition to this phase III trial, Olvi-Vec is currently in phase I trials for the treatment of small-cell and non-small lung cancer. The results of the phase III trial are expected to be announced in the first half of 2026.

The second oncolytic VACV to reach a phase III trial (NCT02562755) is Pexa-Vac (JX-594), developed by SillaJen. JX-594 is engineered with a deletion of the TK gene and insertion of transgenes encoding human GM-CSF and β-galactosidase. It is administered via intratumoral injection. Pexa-Vac has been evaluated in clinical trials for renal, colorectal, and liver cancers, and advanced to a phase III trial for hepatocellular carcinoma in combination with the targeted therapy sorafenib. Unfortunately, this trial failed to demonstrate a survival benefit compared to the control group [1].

Nevertheless, additional trials exploring JX-594 in combination with other therapeutic agents and across different cancer types are still ongoing.

The fact that no VACV-based oncolytic therapies have yet received FDA approval does not mean that they hold less potential than similar approaches based on HSV or adenoviral vectors. First, because of its ability to replicate selectively in cancer cells, VACV is often administered intravenously—a more challenging delivery route compared to the intratumoral injections used for other viruses. Second, it has been tested against highly aggressive and immune-resistant tumors such as liver cancer, pancreatic cancer, and lung cancer, which are generally less responsive to immunotherapy than, for example, melanoma. Finally, the viruses used in earlier unsuccessful trials were first-generation vectors that did not fully exploit VACV’s potential to induce a robust anti-cancer response. As newer generations of oncolytic VACV continue to be developed—with novel inserts and highly effective transgene combinations—more candidates are expected to enter clinical trials, potentially yielding more promising results.

Despite its many advantages, VACV also presents certain challenges that may limit its therapeutic potential. As a replicating virus, it poses a theoretical risk to immunocompromised patients, particularly when administered systemically (e.g., via intravenous injection). However, this risk is significantly mitigated by the presence of multiple attenuating mutations engineered into oncolytic VACV strains, many of which are based on the Lister strain, which has a long history of safe use as a smallpox vaccine.

Another major challenge is the strong immune memory elicited by VACV at both the B and T cell levels. This response can limit the efficacy of repeated administrations, especially through intravenous delivery. High titers of neutralizing antibodies may block the virus from binding to target cells, while cytotoxic memory T cells can destroy infected cancer cells before sufficient viral replication and spread occurs. Because VACV was used as the smallpox vaccine, which was administered globally until 1981, a significant proportion of patients over the age of 50 are likely to retain some level of anti-VACV immunity. In theory, this pre-existing immunity could reduce the efficacy of VACV-based oncolytic therapies in older patients compared to younger individuals who have never been exposed to the smallpox vaccine. For patients with particularly high levels of anti-VACV antibodies, intratumoral injection may be the preferred delivery route. Alternatively, pre-treatment with pharmaceutical agents prior to intravenous injection can help minimize the effects of pre-existing immunity.

Strategies to overcome anti-VACV immune memory have already been developed. For example, treatment with the cyclooxygenase-2 (COX-2) inhibitor celecoxib prior to administering a second viral dose significantly reduced the generation of neutralization antibodies and restored viral titers in pre-immunized models [226]. Similarly, inhibiting the complement system with the synthetic compound CP40 or depleting it with cobra venom factor (CVF) markedly increased viral titers in both blood and tumor tissue of pre-immunized animals [227].

To further protect VACV from complement-mediated neutralization, researchers have engineered a modified virus that expresses the N-terminal fragment of the complement regulatory protein CD55 fused to six membrane proteins found on IMVs. This engineered virus maintained its replication efficiency and infectivity while successfully evading neutralization by VACV-specific antibodies generated after multiple systemic administrations [228].

In conclusion, although pre-existing immunity to VACV poses a significant challenge for repeated dosing, recent advances offer practical strategies to overcome these challenges and enhance the clinical efficacy of oncolytic VACV.

## 9. Conclusions

The limited efficacy of current treatments for disseminated cancer underscores the urgent need for more innovative and potent treatment strategies. VACV has emerged as a highly promising platform for viro-immunotherapy due to its safety, genetic flexibility, and ability to deliver multiple therapeutic genes directly to tumor tissue. It has shown significant potential both as monotherapy and in combination with other treatment modalities.

Although the phase III clinical trial of JX-594 did not yield the desired outcome, it is important to note that this virus represents an earlier generation construct, far less advanced than the more sophisticated candidates currently under development. As our understanding of tumor biology, immunology, and viral engineering continues to evolve, we are poised to design increasingly sophisticated VACV derivatives with improved efficacy and tumor specificity. Moreover, the strategic integration of VACV into combination therapies is expected to become more refined and effective.

With ongoing research and technological advancements, the future of cancer treatment involving VACV appears highly promising and is likely to play a pivotal role in the next generation of oncologic therapies.

## Figures and Tables

**Table 2:** Clinical studies of vaccinia virus as an oncolytic agent published between 2009 and 2025.

Trial# Name, Strain, and Sponsor	Gene Inactivation	Transgene	Phase (Route)	Number of Patients (Cancer Type)	Design	Toxicity	Response	Ref.
**NCT00554372** JX-594 (Pexa-Vec/TG6006) *Wyeth* SillaJen, Inc. Busan, South Korea	TK	GM-CSF *lac*Z	Phase II (Intratumoral)	30 (Advanced Hepatocellular Carcinoma, HCC)	Two groups treated with high dose 1 × 10^9^ PFU and low dose 1 × 10^8^ PFU, respectively	Fever, chills, fatigue, nausea. Also: hypertension in 10% of patients and thrombocytopenia in 7%	62.5% of high-dose group showed tumor necrosis on MRI vs. 35.7% of low-dose group. Median overall survival (OS): 14.1 months for high-dose group and 6.7 months for low-dose group. Historical controls: 6–8 months.	[205]
**NCT01387555** JX-594 (Pexa-Vec/TG6006) *Wyeth* SillaJen, Inc. Busan, South Korea	TK	GM-CSF *lac*Z	Phase IIb (Intravenous infusion + later intratumoral injections)	129 (Advanced HCC, refractory or ineligible to sorafenib)	Two groups: Virus + Best Supportive Care (BSC) vs. BSC alone Pexa-Vec was given as a single intravenous (IV) infusion followed by up to 5 IT injections at 1 × 10^9^ PFU each	Fever, chills, fatigue, nausea. Also: hypertension in 8% of patients and thrombocytopenia in 6%	No statistically significant difference between two groups. Median OS: ~4.2 months (Virus + BSC group) vs. 4.4 months (BSC only) Study terminated early as it did not meet its primary survival endpoint and lacked clear benefits.	[206]
**NCT02562755** JX-594 (Pexa-Vec/TG6006) *Wyeth* SillaJen, Inc. Busan, South Korea	TK	GM-CSF *lac*Z	Phase III (Intratumoral)	459 patients (Advanced HCC)	Three intratumoral doses at 1 × 10^9^ PFU plus sorafenib orally (400 mg BID) vs. sorafenib alone	Pyrexia, diarrhea, decreased appetite, nausea, fatigue	No survival benefit, trial was stopped due to futility after interim analysis. Median OS: ~12.7 months (virus + sorafenib) vs. 14.0 months (sorafenib).	[207]
**NCT02630368** JX-594 (Pexa-Vec/TG6006) *Wyeth* SillaJen, Inc. Busan, South Korea	TK	GM-CSF *lac*Z	Phase II (Intravenous)	20 patients (Soft-tissue sarcoma)	Dose of 1 × 10^9^ PFU every 2 weeks for the first 3 injections and then every 3 weeks combined with low-dose cyclophosphamide, compared to the patients treated with low-dose phosphamide only	Fever and fatigue; single case of grade 3 lymphopenia and grade 3 fever.	Both progression-free survival (PFS) and OS were lower in the JX-594 + cyclophosphamide group vs. the cyclophosphamide-only group.	[208]
**NCT01171651** JX-594 (Pexa-Vec/TG6006) *Wyeth* SillaJen, Inc. Busan, South Korea	TK	GM-CSF *lac*Z	Phase II (Intravenous and intratumoral)	25 patients with unresectable primary HCC	One intravenous dose followed by two intratumoral doses each once a week followed by sorafenib at Day 25	Fever, chills, headache, and nausea	47% of patients displayed responses after treatment with virus only; after including sorafenib, responses were observed in 75% of patients.	[209]
**Unspecified** JX-594 (Pexa-Vec/TG6006) *Wyeth* SillaJen, Inc. Busan, South Korea	TK	GM-CSF *lac*Z	Phase II (Intravenous)	17 patients with with Metastatic, Refractory Renal Cell Carcinoma (RCC).	5 weekly intravenous infusions, followed by extra doses if response is observed	Transient flu-like illness, vomiting, chills, nausea	76% of patients displayed disease control including one complete response.	[210]
**NCT02977156** JX-594 (Pexa-Vec/TG6006) *Wyeth* Transgene Illkirch-Graffenstaden, France	TK	GM-CSF *lac*Z	Phase I (Intravenous and intratumoral)	22 patients with metastatic/advanced solid Tumors	Single intravenous injection at 1 × 10^9^ PFU followed by up to 4 intratumoral injections every week followed by treatment with ipilimumab	No data available	The trial is marked as complete but no information about efficacy has been revealed. Since the asset is no longer in the company’s pipeline, it is likely that efficacy was not satisfactory.	[211]
**NCT03206073** JX-594 (Pexa-Vec/TG6006) *Wyeth* SillaJen, Inc. Busan, South Korea	TK	GM-CSF *lac*Z	Phase I/II (Intravenous and intratumoral)	34 patients with refractory metastatic colorectal cancer	Four doses every 2 weeks at 3 × 10^8^ or 1 × 10^9^ PFU followed by treatment with durvalumab; 18 patients received additional single dose of tremelimumab on day 1	Fever and decreased lymphocyte count	Disease control rate was 12.5% in the group without tremelimumab and 16.7% in the group with tremelimumab. OS was 5.2 months with tremelimumab and 7.5 months without tremelimumab.	[212]
**NCT01443260** GL-ONC1 (GLV-1h68) *Lister* Genelux, Inc. Westlake Village, CA, USA	TK, hemagglutinin, F14.5L	Ruc-GFP, β-glucuronidase, and β-galactosidase	Phase I (Intraperitoneal)	9 patients (7 with peritoneal carcinomatosis and 2 with peritoneal mesothelioma)	Three doses ranging from 10^7^ to 10^9^ PFU per cycle via intraperitoneal infusion	Decrease in lymphocyte count; increase in C-reactive protein (CRP), pyrexia, and abdominal pain	Stable disease observed in ~30% of evaluable patients (no complete/partial responses). Median OS ~5 months vs. 3–6 months in historical controls.	[213]
**NCT00794131** GL-ONC1 (GLV-1h68) *Lister* Genelux, Inc. Westlake Village, CA, USA	TK, hemagglutinin, F14.5L	Ruc-GFP, β-glucuronidase, and β-galactosidase	Phase I (Intravenous)	24 patients with advanced solid tumors (including colorectal, head/neck, and lung tumors)	IIV infusion (dose-escalation study) 3 infusions at doses ranging from 10^5^ to 10^9^ PFU	Pyrexia, musculoskeletal pain, fatigue, nausea.	Stable disease observed in 33% patients, but no partial or complete response.	[214]
**NCT01584284** GL-ONC1 (GLV-1h68) *Lister* Genelux, Inc. Westlake Village, CA, USA	TK, hemagglutinin, F14.5L	Ruc-GFP, β-glucuronidase, and β-galactosidase	Phase I (Intravenous)	19 patients with advanced head and neck carcinoma	3 cohorts of patients treated with single dose at 3 × 10^8^, 1 × 10^9^ and 3 × 10^9^ PFU; 1 cohort treated with 2 doses at 3 × 10^9^ PFU and 1 cohort treated with 4 doses at 3 × 10^9^ PFU every 5–7 days; virus treatment combined with cis-platin and radiotherapy	Nausea, fatigue, mucositis, dysphagia	Favorable results in terms of PFS and OS were observed in the virus treated group compared with historical data.	[215]
**NCT01766739** GL-ONC1 (GLV-1h68) *Lister* Genelux, Inc. Westlake Village, CA, USA	TK, hemagglutinin, F14.5L	Ruc-GFP, β-glucuronidase, and β-galactosidase	Phase I (Intraperitoneal)	18 patients with malignant pleural effusions from mesothelioma, breast, or non-small cell lung cancer	Single doses of 1 × 10^7^, 1 × 10^8^, 1 × 10^9^, or 3 × 10^9^ PFU	Fever, chills, and flu-like symptoms	Virus was detected in tumor tissues; tumor cell density decreased, while density of immune cells increased. Following completion of the trial, patients received subsequent other therapies, so it is hard to precisely determine the effect of virus alone.	[216,217]
**NCT02759588** GL-ONC1 (GLV-1h68) *Lister* Genelux, Inc. Westlake Village, CA, USA	TK, hemagglutinin, F14.5L	Ruc-GFP, β-glucuronidase, and β-galactosidase	Phase II (Intraperitoneal)	27 patients with ovarian cancer	3 × 10^9^ PFU administered on 2 consecutive days followed by platinum doublet chemotherapy with or without bevacizumab	Pyrexia, abdominal pain, and nausea	54% evaluable by RECIST 1.1 had an objective response, with a median PFS of 11.0 months.	[218]
**NCT02714374** GL-ONC1 (GLV-1h68) *Lister* Genelux, Inc. Westlake Village, CA, USA	TK, hemagglutinin, F14.5L	Ruc-GFP, β-glucuronidase, and β-galactosidase	Phase I (Intravenous)	5 patients with solid organ cancers	1 × 10^9^ PFU administered intravenously prior to surgery	Limited data; according to the company’s website, it was well tolerated	Study terminated due to insufficient funding. According to company’s website, virus replication and increased infiltration by immune cells were detected in treated patients.	[219,220]
**NCT03420430** GL-ONC1 (GLV-1h68) *Lister* Genelux, Inc. Westlake Village, CA, USA	TK, hemagglutinin, F14.5L	Ruc-GFP, β-glucuronidase, and β-galactosidase	Expanded access (Intravenous)	4 patients with solid tumors and 6 patients with blood tumors with no standard of care treatment available	IV administration; dose not officially revealed.	Limited data; according to the company’s website, it was well tolerated	No official results posted.	[219,221]
**NCT03294486** TG6002 *Copenhagen* Transgene Illkirch-Graffenstaden, France	TK, RR	cytosine deaminase and uracil phosphoribosyl transferase	Phase I/IIa (Intravenous)	78 patients with recurrent glioblastoma	3 weekly IV infusions at the dose of 1 × 10^5^ PFU combined with fluorocytosine (5-FC)	Limited data; according to the company, toxicity was low and included usual mild symptoms (fever, fatigue, etc.)	Limited data. According to the official company website, the results were favorable. However, the asset was dropped from the pipeline, suggesting that efficacy was not high enough.	[222]
**NCT03724071** TG6002 *Copenhagen* Transgene Illkirch-Graffenstaden, France	TK, RR	cytosine deaminase and uracil phosphoribosyl transferase	Phase I (Intravenous)	15 patients with gastric carcinoma	3 infusions once a week at different doses 3 × 10^8^, 1 × 10^9^ and 3 × 10^9^ PFU combined with 5-Fluorocytosine	None of the patients displayed signs of vaccinia-induced disease.	Virus and transgene expression were detected in tumors. No other data available probably due to low efficacy. TG6002 is no longer in the company’s pipeline.	[223]
**Eudra-CT 2018-004103-39** TG6002 Copenhagen Transgene Illkirch-Graffenstaden, France	TK, RR	cytosine deaminase and uracil phosphoribosyl transferase	Phase I (Intrahepatic artery delivery)	15 patients with Liver-Dominant Metastatic Colorectal Cancer	Two infusions 43 days apart with the dose range from 1 × 10^6^ to 1 × 10^9^ PFU combined with 5-fluorocytosine	Gastrointestinal disorders, pyrexia, fatigue.	Virus and transgene expression were detected in tumors No patients had an objective response based on a 10-week disease control rate.	[224]
**Unspecified** *vvDD* *Western Reserve* NCI Bethesda, MD, USA	TK, VGF	NA	Phase I (Intratumoral)	17 patients with advanced solid tumors	Single injections of the virus with the dose range from 3 × 10^7^ to 3 × 10^9^ PFU	Fever, nausea, fatigue	Virus replication detected in tumors, but true clinical benefit was not achieved in any of the patients.	[36]
**NCT04301011** Tbio-6517 *Copenhagen* Turnstone Biologics San Diego, CA, USA	25 kb deletion from the virus genome including 32 genes	anti-CTLA-4 antibody, FLT3 ligand (fms7 -like tyrosine kinase 3 ligand; FLT3L), and membrane-bound IL-12 (p35 subunit)	Phase I/IIa (Intratumoral or intravenous)	27 Patients with advanced solid tumors	Administered intratumorally or intravenously Alone and in combination with Pembrolizumab; no info on dose revealed	Lack of information	Study was terminated and the asset was withdrawn from pipeline. Official reason not revealed.	[225]

**Table 3:** List of currently (as of July 2025) running clinical trials involving different modifications of vaccinia virus according to https://clinicaltrials.gov/.

Trail Number	Name of the Asset	Sponsor	Phase	Type of Cancer	Number of Patients	Viral Transgenes	Route
NCT07006077	hV01	Hangzhou Converd Co., Ltd., Hangzhou, China	2	Pancreatic	12	IL-21	Intratumoral
NCT06910657	IDOV-Immune	ViroMissile, Inc, La Jolla, CA, USA	1	Advanced solid tumors	78	Unknown	Intravenous
NCT05914376	hV01	Hangzhou Converd Co., Ltd., Hangzhou, China	1	Advanced solid tumors	24	IL-21	Intratumoral
NCT06508307	GC001	GONGCHU Biotechnology Co., Ltd., Hangzhou, China	1	Advanced solid tumors	21	Unknown	Intratumoral
NCT04887025	RGV004	Second Affiliated Hospital, School of Medicine, Zhejiang University, Hangzhou, China	1	B cell lymphoma	25	CD19/CD3 bispecific antibody	Intratumoral
NCT06660056	GC001	GONGCHU Biotechnology Co., Ltd., Hangzhou, China	1	Glioma	35	Unknown	Intratumoral
NCT05684731	KM1	Tongji Hospital, Shanghai, China	1	Ovarian cancer	30	Unknown	Intraperitoneal
NCT07001592	vvDD-hIL2	Allegheny Singer Research Institute, Pittsburgh, PA, USA	1	Advanced abdominal cancer	18	IL-2	Intratumoral
NCT06380309	IDOV-Safe	Peking University, Beijing, China	1	Advanced solid tumors	60	Unknown	Intravenous
NCT04226066	T601	Tasly Tianjin Biopharmaceutical Co., Tianjin, China	1, 2	Advanced solid tumors	69	Cytosine deaminase/uracil phosphoribosyl transferase	Intravenous
NCT04849260	Pexa-Vec	Lee’s Pharmaceutical Limited, Shatin, Hong Kong	1, 2	Melanoma	54	GM-CSF, lacZ	Intratumoral
NCT06463665	Olvi-Vec (GL-ONC1)	Genelux Corporation, Westlake Village, CA, USA	2	Non-small-cell lung cancer	142	Ruc-GFP, β-glucuronidase, and β-galactosidase	Intravenous
NCT05788926	TG6050	Transgene, Illkirch-Graffenstaden, France	1	Non-small-cell lung cancer	36	IL-12 and anti-CTLA4 antibody	Intravenous
NCT05859074	MQ710	Memorial Sloan Kettering Cancer Center, New York, NY, USA	1	Solid tumors	5	Flt3l, OX40L	Intratumoral
NCT05281471	Olvi-Vec (GL-ONC1)	Genelux Corporation, Westlake Village, CA, USA	3	Ovarian cancer	186	Ruc-GFP, β-glucuronidase, and β-galactosidase	Intraperitoneal
NCT04725331	BT001	Transgene, Illkirch-Graffenstaden, France	1, 2	Metastatic or advanced solid tumors	48	GM-CSF, and anti-CTLA4 antibody	Intratumoral
NCT06444815	VET3-TGI	KaliVir Immunotherapeutics, Pittsburgh, PA, USA	1	Advanced solid tumors	60	IL-12 and TGF-β inhibitor	Intravenous or Intratumoral
NCT05346484	CF33-hNIS (VAXINIA)	Imugene Limited, Sydney, Australia	1	Metastatic or advanced solid tumors	100	Human sodium iodide symporter	Intratumoral or Intravenous
NCT05081492	CF33-hNIS-antiPDL1	City of Hope Medical Center, Duarte, CA, USA	1	Triple-negative breast cancer	9	Human sodium iodide symporter and anti-PD-L1 antibody	Intratumoral
NCT06063317	CF33-CD19	Imugene Limited, Sydney, Australia	1	Metastatic or advanced solid tumors	50	Truncated version of human CD19	Intratumoral or Intravenous
NCT06346041	IDOV-Safe	Cancer Institute and Hospital, Chinese Academy of Medical Sciences, Beijing, China	1	Advanced solid tumors	19	Unknown	Intravenous
NCT06713148	IDOV-Safe	Fudan University, Shanghai, China	1	Advanced solid tumors	42	Unknown	Intravenous
NCT03294083	Pexa-Vec	SillaJen, Inc., Busan, South Korea	1, 2	Renal Cell Carcinoma	89	GM-CSF, lacZ	Intratumoral or Intravenous

## References

[1] Xu L., Sun H., Lemoine N.R., Xuan Y., Wang P. (2024). Oncolytic Vaccinia Virus and Cancer Immunotherapy. Front. Immunol..

[2] Kaynarcalidan O., Moreno Mascaraque S., Drexler I. (2021). Vaccinia Virus: From Crude Smallpox Vaccines to Elaborate Viral Vector Vaccine Design. Biomedicines.

[3] Greseth M.D., Czarnecki M.W., Bluma M.S., Traktman P. (2018). Isolation and Characterization of vΔI3 Confirm That Vaccinia Virus SSB Plays an Essential Role in Viral Replication. J. Virol..

[4] Wittek R., Granoff A., Webster R.G. (1999). Vaccinia Virus (*Poxviridae*). Encyclopedia of Virology.

[5] Molteni C., Forni D., Cagliani R., Clerici M., Sironi M. (2022). Genetic Ancestry and Population Structure of Vaccinia Virus. NPJ Vaccines.

[6] Moss B. (2013). Poxvirus DNA Replication. Cold Spring Harb. Perspect. Biol..

[7] Yang Z., Maruri-Avidal L., Sisler J., Stuart C.A., Moss B. (2013). Cascade Regulation of Vaccinia Virus Gene Expression Is Modulated by Multistage Promoters. Virology.

[8] Chou W., Ngo T., Gershon P.D. (2012). An Overview of the Vaccinia Virus Infectome: A Survey of the Proteins of the Poxvirus-Infected Cell. J. Virol..

[9] Laudermilch E., Chandran K. (2021). MAVERICC: Marker-Free Vaccinia Virus Engineering of Recombinants through in Vitro CRISPR/Cas9 Cleavage. J. Mol. Biol..

[10] Jacobs B.L., Langland J.O., Kibler K.V., Denzler K.L., White S.D., Holechek S.A., Wong S., Huynh T., Baskin C.R. (2009). Vaccinia Virus Vaccines: Past, Present and Future. Antivir. Res..

[11] de Freitas L.F.D., Oliveira R.P., Miranda M.C.G., Rocha R.P., Barbosa-Stancioli E.F., Faria A.M.C., da Fonseca F.G. (2019). The Virulence of Different Vaccinia Virus Strains Is Directly Proportional to Their Ability To Downmodulate Specific Cell-Mediated Immune Compartments In Vivo. J. Virol..

[12] Belongia E.A., Naleway A.L. (2003). Smallpox Vaccine: The Good, the Bad, and the Ugly. Clin. Med. Res..

[13] Meiser A., Boulanger D., Sutter G., Krijnse Locker J. (2003). Comparison of Virus Production in Chicken Embryo Fibroblasts Infected with the WR, IHD-J and MVA Strains of Vaccinia Virus: IHD-J Is Most Efficient in Trans-Golgi Network Wrapping and Extracellular Enveloped Virus Release. J. Gen. Virol..

[14] Bengali Z., Townsley A.C., Moss B. (2009). Vaccinia Virus Strain Differences in Cell Attachment and Entry. Virology.

[15] Carter G.C., Law M., Hollinshead M., Smith G.L. (2005). Entry of the Vaccinia Virus Intracellular Mature Virion and Its Interactions with Glycosaminoglycans. J. Gen. Virol..

[16] MacLeod D.T., Nakatsuji T., Wang Z., di Nardo A., Gallo R.L. (2015). Vaccinia Virus Binds to the Scavenger Receptor MARCO on the Surface of Keratinocytes. J. Investig. Dermatol..

[17] Laliberte J.P., Weisberg A.S., Moss B. (2011). The Membrane Fusion Step of Vaccinia Virus Entry Is Cooperatively Mediated by Multiple Viral Proteins and Host Cell Components. PLoS Pathog..

[18] Townsley A.C., Weisberg A.S., Wagenaar T.R., Moss B. (2006). Vaccinia Virus Entry into Cells via a Low-pH-Dependent Endosomal Pathway. J. Virol..

[19] Mercer J., Knébel S., Schmidt F.I., Crouse J., Burkard C., Helenius A. (2010). Vaccinia Virus Strains Use Distinct Forms of Macropinocytosis for Host-Cell Entry. Proc. Natl. Acad. Sci. USA.

[20] Greseth M.D., Traktman P. (2022). The Life Cycle of the Vaccinia Virus Genome. Annu. Rev. Virol..

[21] Schmidt F.I., Bleck C.K.E., Reh L., Novy K., Wollscheid B., Helenius A., Stahlberg H., Mercer J. (2013). Vaccinia Virus Entry Is Followed by Core Activation and Proteasome-Mediated Release of the Immunomodulatory Effector VH1 from Lateral Bodies. Cell Rep..

[22] Tolonen N., Doglio L., Schleich S., Locker J.K. (2001). Vaccinia Virus DNA Replication Occurs in Endoplasmic Reticulum-Enclosed Cytoplasmic Mini-Nuclei. MBoC.

[23] Moss B., Carrasco L., Sonenberg N., Wimmer E. (1993). Cascade Regulation of Vaccinia Virus Gene Expression. Regulation of Gene Expression in Animal Viruses.

[24] Howell L.M., Gracie N.P., Newsome T.P. (2024). Single-Cell Analysis of VACV Infection Reveals Pathogen-Driven Timing of Early and Late Phases and Host-Limited Dynamics of Virus Production. PLoS Pathog..

[25] Liu L., Cooper T., Howley P.M., Hayball J.D. (2014). From Crescent to Mature Virion: Vaccinia Virus Assembly and Maturation. Viruses.

[26] Boyle K.A., Stanitsa E.S., Greseth M.D., Lindgren J.K., Traktman P. (2011). Evaluation of the Role of the Vaccinia Virus Uracil DNA Glycosylase and A20 Proteins as Intrinsic Components of the DNA Polymerase Holoenzyme. J. Biol. Chem..

[27] Rochester S.C., Traktman P. (1998). Characterization of the Single-Stranded DNA Binding Protein Encoded by the Vaccinia Virus I3 Gene. J. Virol..

[28] Garcia A.D., Moss B. (2001). Repression of Vaccinia Virus Holliday Junction Resolvase Inhibits Processing of Viral DNA into Unit-Length Genomes. J. Virol..

[29] Paran N., De Silva F.S., Senkevich T.G., Moss B. (2009). Cellular DNA Ligase I Is Recruited to Cytoplasmic Vaccinia Virus Factories and Masks the Role of the Vaccinia Ligase in Viral DNA Replication. Cell Host Microbe.

[30] El Omari K., Solaroli N., Karlsson A., Balzarini J., Stammers D.K. (2006). Structure of Vaccinia Virus Thymidine Kinase in Complex with dTTP: Insights for Drug Design. BMC Struct. Biol..

[31] Topalis D., Collinet B., Gasse C., Dugué L., Balzarini J., Pochet S., Deville-Bonne D. (2005). Substrate Specificity of Vaccinia Virus Thymidylate Kinase. FEBS J..

[32] Gammon D.B., Gowrishankar B., Duraffour S., Andrei G., Upton C., Evans D.H. (2010). Vaccinia Virus–Encoded Ribonucleotide Reductase Subunits Are Differentially Required for Replication and Pathogenesis. PLoS Pathog..

[33] Smith G.L., Vanderplasschen A., Law M. (2002). The Formation and Function of Extracellular Enveloped Vaccinia Virus. J. Gen. Virol..

[34] Roberts K.L., Smith G.L. (2008). Vaccinia Virus Morphogenesis and Dissemination. Trends Microbiol..

[35] Smith G.L., Law M. (2004). The Exit of Vaccinia Virus from Infected Cells. Virus Res..

[36] Zeh H.J., Downs-Canner S., McCart J.A., Guo Z.S., Rao U.N.M., Ramalingam L., Thorne S.H., Jones H.L., Kalinski P., Wieckowski E. (2015). First-in-Man Study of Western Reserve Strain Oncolytic Vaccinia Virus: Safety, Systemic Spread, and Antitumor Activity. Mol. Ther..

[37] Schwanke H., Stempel M., Brinkmann M.M. (2020). Of Keeping and Tipping the Balance: Host Regulation and Viral Modulation of IRF3-Dependent IFNB1 Expression. Viruses.

[38] Cell Fate in Antiviral Response Arises in the Crosstalk of IRF, NF-κB and JAK/STAT Pathways|Nature Communications. https://www.nature.com/articles/s41467-017-02640-8.

[39] El-Jesr M., Teir M., Maluquer de Motes C. (2020). Vaccinia Virus Activation and Antagonism of Cytosolic DNA Sensing. Front. Immunol..

[40] Peters N.E., Ferguson B.J., Mazzon M., Fahy A.S., Krysztofinska E., Arribas-Bosacoma R., Pearl L.H., Ren H., Smith G.L. (2013). A Mechanism for the Inhibition of DNA-PK-Mediated DNA Sensing by a Virus. PLoS Pathog..

[41] Scutts S.R., Ember S.W., Ren H., Ye C., Lovejoy C.A., Mazzon M., Veyer D.L., Sumner R.P., Smith G.L. (2018). DNA-PK Is Targeted by Multiple Vaccinia Virus Proteins to Inhibit DNA Sensing. Cell Rep..

[42] Chiu Y.-H., MacMillan J.B., Chen Z.J. (2009). RNA Polymerase III Detects Cytosolic DNA and Induces Type I Interferons through the RIG-I Pathway. Cell.

[43] Valentine R., Smith G.L. (2010). Inhibition of the RNA Polymerase III-Mediated dsDNA-Sensing Pathway of Innate Immunity by Vaccinia Virus Protein E3. J. Gen. Virol..

[44] Unterholzner L., Keating S.E., Baran M., Horan K.A., Jensen S.B., Sharma S., Sirois C.M., Jin T., Latz E., Xiao T.S. (2010). IFI16 Is an Innate Immune Sensor for Intracellular DNA. Nat. Immunol..

[45] Almine J.F., O’Hare C.A.J., Dunphy G., Haga I.R., Naik R.J., Atrih A., Connolly D.J., Taylor J., Kelsall I.R., Bowie A.G. (2017). IFI16 and cGAS Cooperate in the Activation of STING during DNA Sensing in Human Keratinocytes. Nat. Commun..

[46] Ablasser A., Schmid-Burgk J.L., Hemmerling I., Horvath G.L., Schmidt T., Latz E., Hornung V. (2013). Cell Intrinsic Immunity Spreads to Bystander Cells via the Intercellular Transfer of cGAMP. Nature.

[47] Luteijn R.D., Zaver S.A., Gowen B.G., Wyman S.K., Garelis N.E., Onia L., McWhirter S.M., Katibah G.E., Corn J.E., Woodward J.J. (2019). SLC19A1 Transports Immunoreactive Cyclic Dinucleotides. Nature.

[48] Seo G.J., Yang A., Tan B., Kim S., Liang Q., Choi Y., Yuan W., Feng P., Park H.-S., Jung J.U. (2015). Akt Kinase-Mediated Checkpoint of cGAS DNA Sensing Pathway. Cell Rep..

[49] Meade N., Furey C., Li H., Verma R., Chai Q., Rollins M.G., DiGiuseppe S., Naghavi M.H., Walsh D. (2018). Poxviruses Evade Cytosolic Sensing through Disruption of an mTORC1-mTORC2 Regulatory Circuit. Cell.

[50] Yang N., Wang Y., Dai P., Li T., Zierhut C., Tan A., Zhang T., Xiang J.Z., Ordureau A., Funabiki H. (2023). Vaccinia E5 Is a Major Inhibitor of the DNA Sensor cGAS. Nat. Commun..

[51] Eaglesham J.B., Pan Y., Kupper T.S., Kranzusch P.J. (2019). Viral and Metazoan Poxins Are cGAMP-Specific Nucleases That Restrict cGAS-STING Signaling. Nature.

[52] Unterholzner L., Sumner R.P., Baran M., Ren H., Mansur D.S., Bourke N.M., Randow F., Smith G.L., Bowie A.G. (2011). Vaccinia Virus Protein C6 Is a Virulence Factor That Binds TBK-1 Adaptor Proteins and Inhibits Activation of IRF3 and IRF7. PLoS Pathog..

[53] Schröder M., Baran M., Bowie A.G. (2008). Viral Targeting of DEAD Box Protein 3 Reveals Its Role in TBK1/IKKε-Mediated IRF Activation. EMBO J..

[54] Ferguson B.J., Benfield C.T.O., Ren H., Lee V.H., Frazer G.L., Strnadova P., Sumner R.P., Smith G.L. (2013). Vaccinia Virus Protein N2 Is a Nuclear IRF3 Inhibitor That Promotes Virulence. J. Gen. Virol..

[55] Willis K.L., Langland J.O., Shisler J.L. (2011). Viral Double-Stranded RNAs from Vaccinia Virus Early or Intermediate Gene Transcripts Possess PKR Activating Function, Resulting in NF-κB Activation, When the K1 Protein Is Absent or Mutated. J. Biol. Chem..

[56] García M.A., Gil J., Ventoso I., Guerra S., Domingo E., Rivas C., Esteban M. (2006). Impact of Protein Kinase PKR in Cell Biology: From Antiviral to Antiproliferative Action. Microbiol. Mol. Biol. Rev..

[57] Szczerba M., Subramanian S., Trainor K., McCaughan M., Kibler K.V., Jacobs B.L. (2022). Small Hero with Great Powers: Vaccinia Virus E3 Protein and Evasion of the Type I IFN Response. Biomedicines.

[58] Cao J., Varga J., Deschambault Y. (2020). Poxvirus Encoded eIF2α Homolog, K3 Family Proteins, Is a Key Determinant of Poxvirus Host Species Specificity. Virology.

[59] Yu H., Bruneau R.C., Brennan G., Rothenburg S. (2021). Battle Royale: Innate Recognition of Poxviruses and Viral Immune Evasion. Biomedicines.

[60] Rivas C., Gil J., Mělková Z., Esteban M., Díaz-Guerra M. (1998). Vaccinia Virus E3L Protein Is an Inhibitor of the Interferon (IFN)-Induced 2-5A Synthetase Enzyme. Virology.

[61] Chan Y.K., Gack M.U. (2015). RIG-I-like Receptor Regulation in Virus Infection and Immunity. Curr. Opin. Virol..

[62] Deng L., Dai P., Parikh T., Cao H., Bhoj V., Sun Q., Chen Z., Merghoub T., Houghton A., Shuman S. (2008). Vaccinia Virus Subverts a Mitochondrial Antiviral Signaling Protein-Dependent Innate Immune Response in Keratinocytes through Its Double-Stranded RNA Binding Protein, E3. J. Virol..

[63] Botos I., Segal D.M., Davies D.R. (2011). The Structural Biology of Toll-like Receptors. Structure.

[64] El-Zayat S.R., Sibaii H., Mannaa F.A. (2019). Toll-like Receptors Activation, Signaling, and Targeting: An Overview. Bull. Natl. Res. Cent..

[65] Harte M.T., Haga I.R., Maloney G., Gray P., Reading P.C., Bartlett N.W., Smith G.L., Bowie A., O’Neill L.A.J. (2003). The Poxvirus Protein A52R Targets Toll-like Receptor Signaling Complexes to Suppress Host Defense. J. Exp. Med..

[66] Stack J., Haga I.R., Schröder M., Bartlett N.W., Maloney G., Reading P.C., Fitzgerald K.A., Smith G.L., Bowie A.G. (2005). Vaccinia Virus Protein A46R Targets Multiple Toll-like–Interleukin-1 Receptor Adaptors and Contributes to Virulence. J. Exp. Med..

[67] Dempsey A., Keating S.E., Carty M., Bowie A.G. (2018). Poxviral Protein E3–Altered Cytokine Production Reveals That DExD/H-Box Helicase 9 Controls Toll-like Receptor–Stimulated Immune Responses. J. Biol. Chem..

[68] Dai P., Cao H., Merghoub T., Avogadri F., Wang W., Parikh T., Fang C.-M., Pitha P.M., Fitzgerald K.A., Rahman M.M. (2011). Myxoma Virus Induces Type I Interferon Production in Murine Plasmacytoid Dendritic Cells via a TLR9/MyD88-, IRF5/IRF7-, and IFNAR-Dependent Pathway. J. Virol..

[69] DiPerna G., Stack J., Bowie A.G., Boyd A., Kotwal G., Zhang Z., Arvikar S., Latz E., Fitzgerald K.A., Marshall W.L. (2004). Poxvirus Protein N1L Targets the I-κB Kinase Complex, Inhibits Signaling to NF-κB by the Tumor Necrosis Factor Superfamily of Receptors, and Inhibits NF-κB and IRF3 Signaling by Toll-like Receptors. J. Biol. Chem..

[70] Albarnaz J.D., Ren H., Torres A.A., Shmeleva E.V., Melo C.A., Bannister A.J., Brember M.P., Chung B.Y.-W., Smith G.L. (2022). Molecular Mimicry of NF-κB by Vaccinia Virus Protein Enables Selective Inhibition of Antiviral Responses. Nat. Microbiol..

[71] Mansur D.S., de Motes C.M., Unterholzner L., Sumner R.P., Ferguson B.J., Ren H., Strnadova P., Bowie A.G., Smith G.L. (2013). Poxvirus Targeting of E3 Ligase β-TrCP by Molecular Mimicry: A Mechanism to Inhibit NF-κB Activation and Promote Immune Evasion and Virulence. PLoS Pathog..

[72] Smith G.L., Benfield C.T.O., Maluquer de Motes C., Mazzon M., Ember S.W.J., Ferguson B.J., Sumner R.P. (2013). Vaccinia Virus Immune Evasion: Mechanisms, Virulence and Immunogenicity. J. Gen. Virol..

[73] Symons J.A., Alcamí A., Smith G.L. (1995). Vaccinia Virus Encodes a Soluble Type I Interferon Receptor of Novel Structure and Broad Species Specificity. Cell.

[74] Mann B.A., Huang J.H., Li P., Chang H.-C., Slee R.B., O’Sullivan A., Mathur A., Yeh N., Klemsz M.J., Brutkiewicz R.R. (2008). Vaccinia Virus Blocks Stat1-Dependent and Stat1-Independent Gene Expression Induced by Type I and Type II Interferons. J. Interferon Cytokine Res..

[75] Alvarez-de Miranda F.J., Alonso-Sánchez I., Alcamí A., Hernaez B. (2021). TNF Decoy Receptors Encoded by Poxviruses. Pathogens.

[76] Symons J.A., Tscharke D.C., Price N., Smith G.L. (2002). A Study of the Vaccinia Virus Interferon-Gamma Receptor and Its Contribution to Virus Virulence. J. Gen. Virol..

[77] Reading P.C., Smith G.L. (2003). Vaccinia Virus Interleukin-18-Binding Protein Promotes Virulence by Reducing Gamma Interferon Production and Natural Killer and T-Cell Activity. J. Virol..

[78] Alcami A., Smith G.L. (1992). A Soluble Receptor for Interleukin-1β Encoded by Vaccinia Virus: A Novel Mechanism of Virus Modulation of the Host Response to Infection. Cell.

[79] Charles A., Janeway J., Travers P., Walport M., Shlomchik M.J. (2001). The Complement System and Innate Immunity. Immunobiology: The Immune System in Health and Disease.

[80] Bernet J., Mullick J., Panse Y., Parab P.B., Sahu A. (2004). Kinetic Analysis of the Interactions between Vaccinia Virus Complement Control Protein and Human Complement Proteins C3b and C4b. J. Virol..

[81] Li P., Wang N., Zhou D., Yee C.S.K., Chang C.-H., Brutkiewicz R.R., Blum J.S. (2005). Disruption of MHC Class II-Restricted Antigen Presentation by Vaccinia Virus1. J. Immunol..

[82] Rehm K.E., Connor R.F., Jones G.J.B., Yimbu K., Roper R.L. (2010). Vaccinia Virus A35R Inhibits MHC Class II Antigen Presentation. Virology.

[83] Orzalli M.H., Kagan J.C. (2017). Apoptosis and Necroptosis as Host Defense Strategies to Prevent Viral Infection. Trends Cell Biol..

[84] Gregory C.D., Devitt A. (2004). The Macrophage and the Apoptotic Cell: An Innate Immune Interaction Viewed Simplistically?. Immunology.

[85] Veyer D.L., Carrara G., Maluquer de Motes C., Smith G.L. (2017). Vaccinia Virus Evasion of Regulated Cell Death. Immunol. Lett..

[86] Maluquer de Motes C., Cooray S., Ren H., Almeida G.M.F., McGourty K., Bahar M.W., Stuart D.I., Grimes J.M., Graham S.C., Smith G.L. (2011). Inhibition of Apoptosis and NF-κB Activation by Vaccinia Protein N1 Occur via Distinct Binding Surfaces and Make Different Contributions to Virulence. PLoS Pathog..

[87] Liu Y., Pan R., Ouyang Y., Gu W., Xiao T., Yang H., Tang L., Wang H., Xiang B., Chen P. (2024). Pyroptosis in Health and Disease: Mechanisms, Regulation and Clinical Perspective. Sig. Transduct. Target. Ther..

[88] Gerlic M., Faustin B., Postigo A., Yu E.C.-W., Proell M., Gombosuren N., Krajewska M., Flynn R., Croft M., Way M. (2013). Vaccinia Virus F1L Protein Promotes Virulence by Inhibiting Inflammasome Activation. Proc. Natl. Acad. Sci. USA.

[89] Liu Y., Liu T., Lei T., Zhang D., Du S., Girani L., Qi D., Lin C., Tong R., Wang Y. (2019). RIP1/RIP3-Regulated Necroptosis as a Target for Multifaceted Disease Therapy (Review). Int. J. Mol. Med..

[90] Koehler H.S., Jacobs B.L. (2023). Subversion of Programed Cell Death by Poxviruses. Curr. Top. Microbiol. Immunol..

[91] Chahroudi A., Chavan R., Koyzr N., Waller E.K., Silvestri G., Feinberg M.B. (2005). Vaccinia Virus Tropism for Primary Hematolymphoid Cells Is Determined by Restricted Expression of a Unique Virus Receptor. J. Virol..

[92] Engelmayer J., Larsson M., Subklewe M., Chahroudi A., Cox W.I., Steinman R.M., Bhardwaj N. (1999). Vaccinia Virus Inhibits the Maturation of Human Dendritic Cells: A Novel Mechanism of Immune Evasion1. J. Immunol..

[93] Yates N.L., Alexander-Miller M.A. (2007). Vaccinia Virus Infection of Mature Dendritic Cells Results in Activation of Virus-Specific Naïve CD8+ T Cells: A Potential Mechanism for Direct Presentation. Virology.

[94] Yao Y., Li P., Singh P., Thiele A.T., Wilkes D.S., Renukaradhya G.J., Brutkiewicz R.R., Travers J.B., Luker G.D., Hong S.-C. (2007). Vaccinia Virus Infection Induces Dendritic Cell Maturation but Inhibits Antigen Presentation by MHC Class II. Cell Immunol..

[95] Byrd D., Shepherd N., Lan J., Hu N., Amet T., Yang K., Desai M., Yu Q. (2014). Primary Human Macrophages Serve as Vehicles for Vaccinia Virus Replication and Dissemination. J. Virol..

[96] Zhou D., Xu W., Ding X., Guo H., Wang J., Zhao G., Zhang C., Zhang Z., Wang Z., Wang P. (2024). Transient Inhibition of Neutrophil Functions Enhances the Antitumor Effect of Intravenously Delivered Oncolytic Vaccinia Virus. Cancer Sci..

[97] Duffy D., Perrin H., Abadie V., Benhabiles N., Boissonnas A., Liard C., Descours B., Reboulleau D., Bonduelle O., Verrier B. (2012). Neutrophils Transport Antigen from the Dermis to the Bone Marrow, Initiating a Source of Memory CD8+ T Cells. Immunity.

[98] Abboud G., Tahiliani V., Desai P., Varkoly K., Driver J., Hutchinson T.E., Salek-Ardakani S. (2015). Natural Killer Cells and Innate Interferon Gamma Participate in the Host Defense against Respiratory Vaccinia Virus Infection. J. Virol..

[99] Dokun A.O., Kim S., Smith H.R., Kang H.S., Chu D.T., Yokoyama W.M. (2001). Specific and Nonspecific NK Cell Activation during Virus Infection. Nat. Immunol..

[100] Chisholm S.E., Reyburn H.T. (2006). Recognition of Vaccinia Virus-Infected Cells by Human Natural Killer Cells Depends on Natural Cytotoxicity Receptors. J. Virol..

[101] Gillard G.O., Bivas-Benita M., Hovav A.-H., Grandpre L.E., Panas M.W., Seaman M.S., Haynes B.F., Letvin N.L. (2011). Thy1+ Nk Cells from Vaccinia Virus-Primed Mice Confer Protection against Vaccinia Virus Challenge in the Absence of Adaptive Lymphocytes. PLoS Pathog..

[102] Shepherd N., Lan J., Li W., Rane S., Yu Q. (2019). Primary Human B Cells at Different Differentiation and Maturation Stages Exhibit Distinct Susceptibilities to Vaccinia Virus Binding and Infection. J. Virol..

[103] Goulding J., Bouge R., Tahiliani V., Croft M., Salek-Ardakani S. (2012). CD8 T Cells Are Essential for Recovery from a Respiratory Vaccinia Virus Infection. J. Immunol..

[104] Bernasconi N.L., Traggiai E., Lanzavecchia A. (2002). Maintenance of Serological Memory by Polyclonal Activation of Human Memory B Cells. Science.

[105] Liu L., Zhong Q., Tian T., Dubin K., Athale S.K., Kupper T.S. (2010). Epidermal Injury and Infection during Poxvirus Immunization Is Crucial for the Generation of Highly Protective T Cell-Mediated Immunity. Nat. Med..

[106] Sánchez-Puig J.M., Sánchez L., Roy G., Blasco R. (2004). Susceptibility of Different Leukocyte Cell Types to Vaccinia Virus Infection. Virol. J..

[107] Hu Z., Molloy M.J., Usherwood E.J. (2016). CD4+ T-Cell Dependence of Primary CD8+ T-Cell Response against Vaccinia Virus Depends upon Route of Infection and Viral Dose. Cell Mol. Immunol..

[108] Dai R., Huang X., Yang Y. (2021). γδT Cells Are Required for CD8+ T Cell Response to Vaccinia Viral Infection. Front. Immunol..

[109] Demkowicz W.E., Littaua R.A., Wang J., Ennis F.A. (1996). Human Cytotoxic T-Cell Memory: Long-Lived Responses to Vaccinia Virus. J. Virol..

[110] Yaghchi C.A., Zhang Z., Alusi G., Lemoine N.R., Wang Y. (2015). Vaccinia Virus, a Promising New Therapeutic Agent for Pancreatic Cancer. Immunotherapy.

[111] Lei W., Ye Q., Hao Y., Chen J., Huang Y., Yang L., Wang S., Qian W. (2022). CD19-Targeted BiTE Expression by an Oncolytic Vaccinia Virus Significantly Augments Therapeutic Efficacy against B-Cell Lymphoma. Blood Cancer J..

[112] Totsch S.K., Schlappi C., Kang K.-D., Ishizuka A.S., Lynn G.M., Fox B., Beierle E.A., Whitley R.J., Markert J.M., Gillespie G.Y. (2019). Oncolytic Herpes Simplex Virus Immunotherapy for Brain Tumors: Current Pitfalls and Emerging Strategies to Overcome Therapeutic Resistance. Oncogene.

[113] Smith G.L., Moss B. (1983). Infectious Poxvirus Vectors Have Capacity for at Least 25 000 Base Pairs of Foreign DNA. Gene.

[114] Gallardo F., Schmitt D., Brandely R., Brua C., Silvestre N., Findeli A., Foloppe J., Top S., Kappler-Gratias S., Quentin-Froignant C. (2020). Fluorescent Tagged Vaccinia Virus Genome Allows Rapid and Efficient Measurement of Oncolytic Potential and Discovery of Oncolytic Modulators. Biomedicines.

[115] Salauddin M., Saha S., Hossain M.G., Okuda K., Shimada M. (2024). Clinical Application of Adenovirus (AdV): A Comprehensive Review. Viruses.

[116] Hiley C.T., Yuan M., Lemoine N.R., Wang Y. (2010). Lister Strain Vaccinia Virus, a Potential Therapeutic Vector Targeting Hypoxic Tumours. Gene Ther..

[117] Ma J., Ramachandran M., Jin C., Quijano-Rubio C., Martikainen M., Yu D., Essand M. (2020). Characterization of Virus-Mediated Immunogenic Cancer Cell Death and the Consequences for Oncolytic Virus-Based Immunotherapy of Cancer. Cell Death Dis..

[118] Fucikova J., Kepp O., Kasikova L., Petroni G., Yamazaki T., Liu P., Zhao L., Spisek R., Kroemer G., Galluzzi L. (2020). Detection of Immunogenic Cell Death and Its Relevance for Cancer Therapy. Cell Death Dis..

[119] O’Leary M.P., Choi A.H., Kim S.-I., Chaurasiya S., Lu J., Park A.K., Woo Y., Warner S.G., Fong Y., Chen N.G. (2018). Novel Oncolytic Chimeric Orthopoxvirus Causes Regression of Pancreatic Cancer Xenografts and Exhibits Abscopal Effect at a Single Low Dose. J. Transl. Med..

[120] Choi A.H., O’Leary M.P., Lu J., Kim S.-I., Fong Y., Chen N.G. (2018). Endogenous Akt Activity Promotes Virus Entry and Predicts Efficacy of Novel Chimeric Orthopoxvirus in Triple-Negative Breast Cancer. Mol. Ther. Oncolytics.

[121] Chaurasiya S., Yang A., Zhang Z., Lu J., Valencia H., Kim S.-I., Woo Y., Warner S.G., Olafsen T., Zhao Y. (2021). A Comprehensive Preclinical Study Supporting Clinical Trial of Oncolytic Chimeric Poxvirus CF33-hNIS-Anti-PD-L1 to Treat Breast Cancer. Mol. Ther. Methods Clin. Dev..

[122] Warner S.G., Kim S.-I., Chaurasiya S., O’Leary M.P., Lu J., Sivanandam V., Woo Y., Chen N.G., Fong Y. (2019). A Novel Chimeric Poxvirus Encoding hNIS Is Tumor-Tropic, Imageable, and Synergistic with Radioiodine to Sustain Colon Cancer Regression. Mol. Ther. Oncolytics.

[123] Yang A., Zhang Z., Chaurasiya S., Park A.K., Jung A., Lu J., Kim S.-I., Priceman S., Fong Y., Woo Y. (2023). Development of the Oncolytic Virus, CF33, and Its Derivatives for Peritoneal-Directed Treatment of Gastric Cancer Peritoneal Metastases. J. Immunother. Cancer.

[124] Chen C., Park A.K., Monroy I., Ren Y., Kim S.-I., Chaurasiya S., Priceman S.J., Fong Y. (2024). Using Oncolytic Virus to Retask CD19-Chimeric Antigen Receptor T Cells for Treatment of Pancreatic Cancer: Toward a Universal Chimeric Antigen Receptor T-Cell Strategy for Solid Tumor. J. Am. Coll. Surg..

[125] Earl P.L., Moss B., Wyatt L.S. (2017). Generation of Recombinant Vaccinia Viruses. Curr. Protoc. Protein Sci..

[126] Domi A., Moss B. (2002). Cloning the Vaccinia Virus Genome as a Bacterial Artificial Chromosome in Escherichia Coli and Recovery of Infectious Virus in Mammalian Cells. Proc. Natl. Acad. Sci. USA.

[127] Yuan M., Zhang W., Wang J., Al Yaghchi C., Ahmed J., Chard L., Lemoine N.R., Wang Y. (2015). Efficiently Editing the Vaccinia Virus Genome by Using the CRISPR-Cas9 System. J. Virol..

[128] Rezaei R., Boulton S., Ahmadi M., Petryk J., Da Silva M., Kooshki Zamani N., Singaravelu R., St-Laurent G., Daniel L., Sadeghipour A. (2025). Antibiotic-Mediated Selection of Randomly Mutagenized and Cytokine-Expressing Oncolytic Viruses. Nat. Biomed. Eng..

[129] Pfeffer C.M., Singh A.T.K. (2018). Apoptosis: A Target for Anticancer Therapy. Int. J. Mol. Sci..

[130] de Queiroz N.M.G.P., Xia T., Konno H., Barber G.N. (2019). Ovarian Cancer Cells Commonly Exhibit Defective STING Signaling Which Affects Sensitivity to Viral Oncolysis. Mol. Cancer Res..

[131] Aye Y., Li M., Long M.J.C., Weiss R.S. (2015). Ribonucleotide Reductase and Cancer: Biological Mechanisms and Targeted Therapies. Oncogene.

[132] Bitter E.E., Townsend M.H., Erickson R., Allen C., O’Neill K.L. (2020). Thymidine Kinase 1 through the Ages: A Comprehensive Review. Cell Biosci..

[133] Potts K.G., Irwin C.R., Favis N.A., Pink D.B., Vincent K.M., Lewis J.D., Moore R.B., Hitt M.M., Evans D.H. (2017). Deletion of F4L (*Ribonucleotide reductase*) in Vaccinia Virus Produces a Selective Oncolytic Virus and Promotes Anti-Tumor Immunity with Superior Safety in Bladder Cancer Models. EMBO Mol. Med..

[134] Puhlmann M., Brown C.K., Gnant M., Huang J., Libutti S.K., Alexander H.R., Bartlett D.L. (2000). Vaccinia as a Vector for Tumor-Directed Gene Therapy: Biodistribution of a Thymidine Kinase-Deleted Mutant. Cancer Gene Ther..

[135] Breitbach C.J., Arulanandam R., De Silva N., Thorne S.H., Patt R., Daneshmand M., Moon A., Ilkow C., Burke J., Hwang T.-H. (2013). Oncolytic Vaccinia Virus Disrupts Tumor-Associated Vasculature in Humans. Cancer Res..

[136] Lai A.C.-K., Pogo B.G.-T. (1989). Attenuated Deletion Mutants of Vaccinia Virus Lacking the Vaccinia Growth Factor Are Defective in Replication in Vivo. Microb. Pathog..

[137] DeHaven B.C., Gupta K., Isaacs S.N. (2011). The Vaccinia Virus A56 Protein: A Multifunctional Transmembrane Glycoprotein That Anchors Two Secreted Viral Proteins. J. Gen. Virol..

[138] Izmailyan R., Chang W. (2008). Vaccinia Virus WR53.5/F14.5 Protein Is a New Component of Intracellular Mature Virus and Is Important for Calcium-Independent Cell Adhesion and Vaccinia Virus Virulence in Mice. J. Virol..

[139] Zhang Q., Liang C., Yu Y.A., Chen N., Dandekar T., Szalay A.A. (2009). The Highly Attenuated Oncolytic Recombinant Vaccinia Virus GLV-1h68: Comparative Genomic Features and the Contribution of F14.5L Inactivation. Mol. Genet. Genom..

[140] McCart J.A., Ward J.M., Lee J., Hu Y., Alexander H.R., Libutti S.K., Moss B., Bartlett D.L. (2001). Systemic Cancer Therapy with a Tumor-Selective Vaccinia Virus Mutant Lacking Thymidine Kinase and Vaccinia Growth Factor Genes. Cancer Res..

[141] Guo Z.S., Naik A., O’Malley M.E., Popovic P., Demarco R., Hu Y., Yin X., Yang S., Zeh H.J., Moss B. (2005). The Enhanced Tumor Selectivity of an Oncolytic Vaccinia Lacking the Host Range and Antiapoptosis Genes SPI-1 and SPI-2. Cancer Res..

[142] Yang S., Guo Z.S., O’Malley M.E., Yin X., Zeh H.J., Bartlett D.L. (2007). A New Recombinant Vaccinia with Targeted Deletion of Three Viral Genes: Its Safety and Efficacy as an Oncolytic Virus. Gene Ther..

[143] Kirn D.H., Wang Y., Le Boeuf F., Bell J., Thorne S.H. (2007). Targeting of Interferon-Beta to Produce a Specific, Multi-Mechanistic Oncolytic Vaccinia Virus. PLoS Med..

[144] Eradication of Solid Human Breast Tumors in Nude Mice with an Intravenously Injected Light-Emitting Oncolytic Vaccinia Virus|Cancer Research|American Association for Cancer Research. https://aacrjournals.org/cancerres/article/67/20/10038/533730/Eradication-of-Solid-Human-Breast-Tumors-in-Nude.

[145] Foloppe J., Kempf J., Futin N., Kintz J., Cordier P., Pichon C., Findeli A., Vorburger F., Quemeneur E., Erbs P. (2019). The Enhanced Tumor Specificity of TG6002, an Armed Oncolytic Vaccinia Virus Deleted in Two Genes Involved in Nucleotide Metabolism. Mol. Ther. Oncolytics.

[146] Jia Y., Wang Y., Zhao G., Yang Y., Yan W., Wang R., Han B., Wang L., Zhang Z., Chen L. (2025). Novel Oncolytic Vaccinia Virus Armed with Interleukin-27 Is a Potential Therapeutic Agent for the Treatment of Murine Pancreatic Cancer. J. Immunother. Cancer.

[147] Neidel S., Torres A.A., Ren H., Smith G.L. (2020). Leaky Scanning Translation Generates a Second A49 Protein That Contributes to Vaccinia Virus Virulence. J. Gen. Virol..

[148] Pelin A., Foloppe J., Petryk J., Singaravelu R., Hussein M., Gossart F., Jennings V.A., Stubbert L.J., Foster M., Storbeck C. (2019). Deletion of Apoptosis Inhibitor F1L in Vaccinia Virus Increases Safety and Oncolysis for Cancer Therapy. Mol. Ther. Oncolytics.

[149] Riederer S., del Canizo A., Navas J., Peter M.G., Link E.K., Sutter G., Rojas J.J. (2023). Improving Poxvirus-Mediated Antitumor Immune Responses by Deleting Viral cGAMP-Specific Nuclease. Cancer Gene Ther..

[150] Papatriantafyllou M. (2011). GM-CSF in Focus. Nat. Rev. Immunol..

[151] (2012). 87. Preclinical and Clinical Evaluation of Oncolytic Immunotherapy with Ad5/3-E2F1-Δ24-GMCSF (CGTG-602), a GM-CSF Producing Adenovirus Targeted to Tumors on Four Levels. Mol. Ther..

[152] Shi X., Sun K., Li L., Xian J., Wang P., Jia F., Xu F. (2024). Oncolytic Activity of Sindbis Virus with the Help of GM-CSF in Hepatocellular Carcinoma. Int. J. Mol. Sci..

[153] Zhang T., Jou T.H.-T., Hsin J., Wang Z., Huang K., Ye J., Yin H., Xing Y. (2023). Talimogene Laherparepvec (T-VEC): A Review of the Recent Advances in Cancer Therapy. J. Clin. Med..

[154] Deng L., Fan J., Guo M., Huang B. (2016). Oncolytic and Immunologic Cancer Therapy with GM-CSF-Armed Vaccinia Virus of Tian Tan Strain Guang9. Cancer Lett..

[155] Xuan Y., Yan W., Wang R., Wang X., Guo Y., Dun H., Huan Z., Xu L., Han R., Sun X. (2025). GM-CSF and IL-21-Armed Oncolytic Vaccinia Virus Significantly Enhances Anti-Tumor Activity and Synergizes with Anti-PD1 Immunotherapy in Pancreatic Cancer. Front. Immunol..

[156] Li J., O’Malley M., Urban J., Sampath P., Guo Z.S., Kalinski P., Thorne S.H., Bartlett D.L. (2011). Chemokine Expression From Oncolytic Vaccinia Virus Enhances Vaccine Therapies of Cancer. Mol. Ther..

[157] Liu Z., Ravindranathan R., Li J., Kalinski P., Guo Z.S., Bartlett D.L. (2016). CXCL11-Armed Oncolytic Poxvirus Elicits Potent Antitumor Immunity and Shows Enhanced Therapeutic Efficacy. OncoImmunology.

[158] Ge Y., Wang H., Ren J., Liu W., Chen L., Chen H., Ye J., Dai E., Ma C., Ju S. (2020). Oncolytic Vaccinia Virus Delivering Tethered IL-12 Enhances Antitumor Effects with Improved Safety. J. Immunother. Cancer.

[159] Shakiba Y., Vorobyev P.O., Yusubalieva G.M., Kochetkov D.V., Zajtseva K.V., Valikhov M.P., Kalsin V.A., Zabozlaev F.G., Semkina A.S., Troitskiy A.V. (2023). Oncolytic Therapy with Recombinant Vaccinia Viruses Targeting the Interleukin-15 Pathway Elicits a Synergistic Response. Mol. Ther.-Oncolytics.

[160] Chen L., Chen H., Ye J., Ge Y., Wang H., Dai E., Ren J., Liu W., Ma C., Ju S. (2021). Intratumoral Expression of Interleukin 23 Variants Using Oncolytic Vaccinia Virus Elicit Potent Antitumor Effects on Multiple Tumor Models via Tumor Microenvironment Modulation. Theranostics.

[161] Chard L.S., Maniati E., Wang P., Zhang Z., Gao D., Wang J., Cao F., Ahmed J., El Khouri M., Hughes J. (2015). A Vaccinia Virus Armed with Interleukin-10 Is a Promising Therapeutic Agent for Treatment of Murine Pancreatic Cancer. Clin. Cancer Res..

[162] Mittal S.K., Cho K.-J., Ishido S., Roche P.A. (2015). Interleukin 10 (IL-10)-Mediated Immunosuppression. J. Biol. Chem..

[163] Holicek P., Guilbaud E., Klapp V., Truxova I., Spisek R., Galluzzi L., Fucikova J. (2024). Type I Interferon and Cancer. Immunol. Rev..

[164] Hirvinen M., Capasso C., Guse K., Garofalo M., Vitale A., Ahonen M., Kuryk L., Vähä-Koskela M., Hemminki A., Fortino V. (2016). Expression of DAI by an Oncolytic Vaccinia Virus Boosts the Immunogenicity of the Virus and Enhances Antitumor Immunity. Mol. Ther.-Oncolytics.

[165] Wang X., Zhou N., Liu T., Jia X., Ye T., Chen K., Li G. (2021). Oncolytic Vaccinia Virus Expressing White-Spotted Charr Lectin Regulates Antiviral Response in Tumor Cells and Inhibits Tumor Growth In Vitro and In Vivo. Mar. Drugs.

[166] Hinterberger M., Giessel R., Fiore G., Graebnitz F., Bathke B., Wennier S., Chaplin P., Melero I., Suter M., Lauterbach H. (2021). Intratumoral Virotherapy with 4-1BBL Armed Modified Vaccinia Ankara Eradicates Solid Tumors and Promotes Protective Immune Memory. J. Immunother. Cancer.

[167] Parviainen S., Ahonen M., Diaconu I., Hirvinen M., Karttunen Å., Vähä-Koskela M., Hemminki A., Cerullo V. (2014). CD40 Ligand and tdTomato-Armed Vaccinia Virus for Induction of Antitumor Immune Response and Tumor Imaging. Gene Ther..

[168] Yang N., Wang Y., Liu S., Tariq S.B., Luna J.M., Mazo G., Tan A., Zhang T., Wang J., Yan W. (2023). OX40L-Expressing Recombinant Modified Vaccinia Virus Ankara Induces Potent Antitumor Immunity via Reprogramming Tregs. J. Exp. Med..

[169] Yu F., Wang X., Guo Z.S., Bartlett D.L., Gottschalk S.M., Song X.-T. (2014). T-Cell Engager-Armed Oncolytic Vaccinia Virus Significantly Enhances Antitumor Therapy. Mol. Ther..

[170] DePeaux K., Rivadeneira D.B., Lontos K., Dean V.G., Gunn W.G., Watson M.J., Yao T., Wilfahrt D., Hinck C., Wieteska L. (2023). An Oncolytic Virus–Delivered TGFβ Inhibitor Overcomes the Immunosuppressive Tumor Microenvironment. J. Exp. Med..

[171] Frentzen A., Yu Y.A., Chen N., Zhang Q., Weibel S., Raab V., Szalay A.A. (2009). Anti-VEGF Single-Chain Antibody GLAF-1 Encoded by Oncolytic Vaccinia Virus Significantly Enhances Antitumor Therapy. Proc. Natl. Acad. Sci. USA.

[172] Brown C.E., Mackall C.L. (2019). CAR T Cell Therapy: Inroads to Response and Resistance. Nat. Rev. Immunol..

[173] Aalipour A., Boeuf F.L., Tang M., Murty S., Simonetta F., Lozano A.X., Shaffer T.M., Bell J.C., Gambhir S.S. (2020). Viral Delivery of CAR Targets to Solid Tumors Enables Effective Cell Therapy. Mol. Ther.-Oncolytics.

[174] Park A.K., Fong Y., Kim S.-I., Yang J., Murad J.P., Lu J., Jeang B., Chang W.-C., Chen N.G., Thomas S.H. (2020). Effective Combination Immunotherapy Using Oncolytic Viruses to Deliver CAR Targets to Solid Tumors. Sci. Transl. Med..

[175] Moon E.K., Wang L.-C.S., Bekdache K., Lynn R.C., Lo A., Thorne S.H., Albelda S.M. (2018). Intra-Tumoral Delivery of CXCL11 via a Vaccinia Virus, but Not by Modified T Cells, Enhances the Efficacy of Adoptive T Cell Therapy and Vaccines. OncoImmunology.

[176] Sivanandam V., LaRocca C.J., Chen N.G., Fong Y., Warner S.G. (2019). Oncolytic Viruses and Immune Checkpoint Inhibition: The Best of Both Worlds. Mol. Ther. Oncolytics.

[177] Chaurasiya S., Chen N.G., Fong Y. (2018). Oncolytic Viruses and Immunity. Curr. Opin. Immunol..

[178] Sun Y., Zhang Z., Zhang C., Zhang N., Wang P., Chu Y., Dunmall L.S.C., Lemoine N.R., Wang Y. (2022). An Effective Therapeutic Regime for Treatment of Glioma Using Oncolytic Vaccinia Virus Expressing IL-21 in Combination with Immune Checkpoint Inhibition. Mol. Ther.-Oncolytics.

[179] Lou J., Dong J., Xu R., Zeng H., Fang L., Wu Y., Liu Y., Wang S. (2021). Remodeling of the Tumor Microenvironment Using an Engineered Oncolytic Vaccinia Virus Improves PD-L1 Inhibition Outcomes. Biosci. Rep..

[180] Liu Z., Ravindranathan R., Kalinski P., Guo Z.S., Bartlett D.L. (2017). Rational Combination of Oncolytic Vaccinia Virus and PD-L1 Blockade Works Synergistically to Enhance Therapeutic Efficacy. Nat. Commun..

[181] Wang G., Kang X., Chen K.S., Jehng T., Jones L., Chen J., Huang X.F., Chen S.-Y. (2020). An Engineered Oncolytic Virus Expressing PD-L1 Inhibitors Activates Tumor Neoantigen-Specific T Cell Responses. Nat. Commun..

[182] Semmrich M., Marchand J.-B., Fend L., Rehn M., Remy C., Holmkvist P., Silvestre N., Svensson C., Kleinpeter P., Deforges J. (2022). Vectorized Treg-Depleting αCTLA-4 Elicits Antigen Cross-Presentation and CD8+ T Cell Immunity to Reject ‘Cold’ Tumors. J. Immunother. Cancer.

[183] Zuo S., Wei M., Xu T., Kong L., He B., Wang S., Wang S., Wu J., Dong J., Wei J. (2021). An Engineered Oncolytic Vaccinia Virus Encoding a Single-Chain Variable Fragment against TIGIT Induces Effective Antitumor Immunity and Synergizes with PD-1 or LAG-3 Blockade. J. Immunother. Cancer.

[184] Advani S.J., Buckel L., Chen N.G., Scanderbeg D.J., Geissinger U., Zhang Q., Yu Y.A., Aguilar R.J., Mundt A.J., Szalay A.A. (2012). Preferential Replication of Systemically Delivered Oncolytic Vaccinia Virus in Focally Irradiated Glioma Xenografts. Clin. Cancer Res..

[185] Storozynsky Q.T., Agopsowicz K.C., Noyce R.S., Bukhari A.B., Han X., Snyder N., Umer B.A., Gamper A.M., Godbout R., Evans D.H. (2023). Radiation Combined with Oncolytic Vaccinia Virus Provides Pronounced Antitumor Efficacy and Induces Immune Protection in an Aggressive Glioblastoma Model. Cancer Lett..

[186] Dai M.H., Liu S.L., Chen N.G., Zhang T.P., You L., Zhang F.Q., Chou T.C., Szalay A.A., Fong Y., Zhao Y.P. (2014). Oncolytic Vaccinia Virus in Combination with Radiation Shows Synergistic Antitumor Efficacy in Pancreatic Cancer. Cancer Lett..

[187] Chen W.-Y., Chen Y.-L., Lin H.-W., Chang C.-F., Huang B.-S., Sun W.-Z., Cheng W.-F. (2021). Stereotactic Body Radiation Combined with Oncolytic Vaccinia Virus Induces Potent Anti-Tumor Effect by Triggering Tumor Cell Necroptosis and DAMPs. Cancer Lett..

[188] Gholami S., Haddad D., Chen C.-H., Chen N.G., Zhang Q., Zanzonico P.B., Szalay A.A., Fong Y. (2011). Novel Therapy for Anaplastic Thyroid Carcinoma Cells Using an Oncolytic Vaccinia Virus Carrying the Human Sodium Iodide Symporter. Surgery.

[189] Gholami S., Chen C.-H., Lou E., De Brot M., Fujisawa S., Chen N.G., Szalay A.A., Fong Y. (2012). Vaccinia Virus GLV-1h153 Is Effective in Treating and Preventing Metastatic Triple-Negative Breast Cancer. Ann. Surg..

[190] Haddad D., Chen N.G., Zhang Q., Chen C.-H., Yu Y.A., Gonzalez L., Carpenter S.G., Carson J., Au J., Mittra A. (2011). Insertion of the Human Sodium Iodide Symporter to Facilitate Deep Tissue Imaging Does Not Alter Oncolytic or Replication Capability of a Novel Vaccinia Virus. J. Transl. Med..

[191] Mansfield D.C., Kyula J.N., Rosenfelder N., Chao-Chu J., Kramer-Marek G., Khan A.A., Roulstone V., McLaughlin M., Melcher A.A., Vile R.G. (2016). Oncolytic Vaccinia Virus as a Vector for Therapeutic Sodium Iodide Symporter Gene Therapy in Prostate Cancer. Gene Ther..

[192] Ottolino-Perry K., Mealiea D., Sellers C., Acuna S.A., Angarita F.A., Okamoto L., Scollard D., Ginj M., Reilly R., McCart J.A. (2023). Vaccinia Virus and Peptide-Receptor Radiotherapy Synergize to Improve Treatment of Peritoneal Carcinomatosis. Mol. Ther.-Oncolytics.

[193] Huang B., Sikorski R., Kirn D.H., Thorne S.H. (2011). Synergistic Anti-Tumor Effects between Oncolytic Vaccinia Virus and Paclitaxel Are Mediated by the IFN Response and HMGB1. Gene Ther..

[194] Yu Y.A., Galanis C., Woo Y., Chen N., Zhang Q., Fong Y., Szalay A.A. (2009). Regression of Human Pancreatic Tumor Xenografts in Mice after a Single Systemic Injection of Recombinant Vaccinia Virus GLV-1h68. Mol. Cancer Ther..

[195] Lun X.Q., Jang J.-H., Tang N., Deng H., Head R., Bell J.C., Stojdl D.F., Nutt C.L., Senger D.L., Forsyth P.A. (2009). Efficacy of Systemically Administered Oncolytic Vaccinia Virotherapy for Malignant Gliomas Is Enhanced by Combination Therapy with Rapamycin or Cyclophosphamide. Clin. Cancer Res..

[196] Hofmann E., Weibel S., Szalay A.A. (2014). Combination Treatment with Oncolytic Vaccinia Virus and Cyclophosphamide Results in Synergistic Antitumor Effects in Human Lung Adenocarcinoma Bearing Mice. J. Transl. Med..

[197] Berchtold S., Beil J., Raff C., Smirnow I., Schell M., D’Alvise J., Gross S., Lauer U.M. (2020). Assessing and Overcoming Resistance Phenomena against a Genetically Modified Vaccinia Virus in Selected Cancer Cell Lines. Int. J. Mol. Sci..

[198] Seubert C.M., Stritzker J., Hess M., Donat U., Sturm J.B., Chen N., von Hof J.M., Krewer B., Tietze L.F., Gentschev I. (2011). Enhanced Tumor Therapy Using Vaccinia Virus Strain GLV-1h68 in Combination with a β-Galactosidase-Activatable Prodrug Seco-Analog of Duocarmycin SA. Cancer Gene Ther..

[199] Lee S., Yang W., Kim D.K., Kim H., Shin M., Choi K.U., Suh D.S., Kim Y.H., Hwang T.-H., Kim J.H. (2022). Inhibition of MEK-ERK Pathway Enhances Oncolytic Vaccinia Virus Replication in Doxorubicin-Resistant Ovarian Cancer. Mol. Ther.-Oncolytics.

[200] Ferguson M.S., Dunmall L.S.C., Gangeswaran R., Marelli G., Tysome J.R., Burns E., Whitehead M.A., Aksoy E., Alusi G., Hiley C. (2020). Transient Inhibition of PI3Kδ Enhances the Therapeutic Effect of Intravenous Delivery of Oncolytic Vaccinia Virus. Mol. Ther..

[201] MacTavish H., Diallo J.-S., Huang B., Stanford M., Boeuf F.L., Silva N.D., Cox J., Simmons J.G., Guimond T., Falls T. (2010). Enhancement of Vaccinia Virus Based Oncolysis with Histone Deacetylase Inhibitors. PLoS ONE.

[202] Kim M., Nitschké M., Sennino B., Murer P., Schriver B.J., Bell A., Subramanian A., McDonald C.E., Wang J., Cha H. (2018). Amplification of Oncolytic Vaccinia Virus Widespread Tumor Cell Killing by Sunitinib through Multiple Mechanisms. Cancer Res..

[203] Heo J., Breitbach C.J., Moon A., Kim C.W., Patt R., Kim M.K., Lee Y.K., Oh S.Y., Woo H.Y., Parato K. (2011). Sequential Therapy With JX-594, A Targeted Oncolytic Poxvirus, Followed by Sorafenib in Hepatocellular Carcinoma: Preclinical and Clinical Demonstration of Combination Efficacy. Mol. Ther..

[204] Chen N.G., and Szalay A.A. (2010). Oncolytic Vaccinia Virus: A Theranostic Agent for Cancer. Future Virol..

[205] Heo J., Reid T., Ruo L., Breitbach C.J., Rose S., Bloomston M., Cho M., Lim H.Y., Chung H.C., Kim C.W. (2013). Randomized Dose-Finding Clinical Trial of Oncolytic Immunotherapeutic Vaccinia JX-594 in Liver Cancer. Nat. Med..

[206] Moehler M., Heo J., Lee H.C., Tak W.Y., Chao Y., Paik S.W., Yim H.J., Byun K.S., Baron A., Ungerechts G. (2019). Vaccinia-Based Oncolytic Immunotherapy Pexastimogene Devacirepvec in Patients with Advanced Hepatocellular Carcinoma after Sorafenib Failure: A Randomized Multicenter Phase IIb Trial (TRAVERSE). Oncoimmunology.

[207] Abou-Alfa G.K., Galle P.R., Chao Y., Erinjeri J., Heo J., Borad M.J., Luca A., Burke J., Pelusio A., Agathon D. (2023). PHOCUS: A Phase 3, Randomized, Open-Label Study of Sequential Treatment with Pexa-Vec (JX-594) and Sorafenib in Patients with Advanced Hepatocellular Carcinoma. Liver Cancer.

[208] Toulmonde M., Cousin S., Kind M., Guegan J.-P., Bessede A., Le Loarer F., Perret R., Cantarel C., Bellera C., Italiano A. (2022). Randomized Phase 2 Trial of Intravenous Oncolytic Virus JX-594 Combined with Low-Dose Cyclophosphamide in Patients with Advanced Soft-Tissue Sarcoma. J. Hematol. Oncol..

[209] Heo J., Breitbach C., Cho M., Hwang T.-H., Kim C.W., Jeon U.B., Woo H.Y., Yoon K.T., Lee J.W., Burke J. (2013). Phase II Trial of Pexa-Vec (Pexastimogene Devacirepvec; JX-594), an Oncolytic and Immunotherapeutic Vaccinia Virus, Followed by Sorafenib in Patients with Advanced Hepatocellular Carcinoma (HCC). JCO.

[210] Kim S.-G., Ha H.K., Lim S., De Silva N.S., Pelusio A., Mun J.H., Patt R.H., Breitbach C.J., Burke J.M. (2018). Phase II Trial of Pexa-Vec (Pexastimogene Devacirepvec; JX-594), an Oncolytic and Immunotherapeutic Vaccinia Virus, in Patients with Metastatic, Refractory Renal Cell Carcinoma (RCC). JCO.

[211] Immunization Strategy with Intra-Tumoral Injections of Pexa-Vec with Ipilimumab in Metastatic/Advanced Solid Tumors. (ISI-JX). https://clinicaltrials.gov/study/NCT02977156.

[212] Monge C., Xie C., Myojin Y., Coffman K., Hrones D.M., Wang S., Hernandez J.M., Wood B.J., Levy E.B., Juburi I. (2023). Phase I/II Study of PexaVec in Combination with Immune Checkpoint Inhibition in Refractory Metastatic Colorectal Cancer. J. Immunother. Cancer.

[213] Lauer U.M., Schell M., Beil J., Berchtold S., Koppenhöfer U., Glatzle J., Königsrainer A., Möhle R., Nann D., Fend F. (2018). Phase I Study of Oncolytic Vaccinia Virus GL-ONC1 in Patients with Peritoneal Carcinomatosis. Clin. Cancer Res..

[214] Pedersen J.V., Karapanagiotou E.M., Biondo A., Tunariu N., Puglisi M., Denholm K.A., Sassi S., Mansfield D., Yap T.A., De Bono J.S. (2011). A Phase I Clinical Trial of a Genetically Modified and Imageable Oncolytic Vaccinia Virus GL-ONC1 with Clinical Green Fluorescent Protein (GFP) Imaging. JCO.

[215] Mell L.K., Brumund K.T., Daniels G.A., Advani S.J., Zakeri K., Wright M.E., Onyeama S.-J., Weisman R.A., Sanghvi P.R., Martin P.J. (2017). Phase I Trial of Intravenous Oncolytic Vaccinia Virus (GL-ONC1) with Cisplatin and Radiotherapy in Patients with Locoregionally Advanced Head and Neck Carcinoma. Clin. Cancer Res..

[216] Krug L.M., Zauderer M.G., Adusumili P.S., McGee E., Sepkowitz K., Klang M., Yu Y.A., Scigalla P., Rusch V.W. (2015). Phase I Study of Intra-Pleural Administration of GL-ONC1, an Oncolytic Vaccinia Virus, in Patients with Malignant Pleural Effusion. JCO.

[217] Chintala N.K., Choe J.K., McGee E., Bellis R., Saini J.K., Banerjee S., Moreira A.L., Zauderer M.G., Adusumilli P.S., Rusch V.W. (2023). Correlative Analysis from a Phase I Clinical Trial of Intrapleural Administration of Oncolytic Vaccinia Virus (Olvi-Vec) in Patients with Malignant Pleural Mesothelioma. Front. Immunol..

[218] Holloway R.W., Mendivil A.A., Kendrick J.E., Abaid L.N., Brown J.V., LeBlanc J., McKenzie N.D., Mori K.M., Ahmad S. (2023). Clinical Activity of Olvimulogene Nanivacirepvec–Primed Immunochemotherapy in Heavily Pretreated Patients With Platinum-Resistant or Platinum-Refractory Ovarian Cancer. JAMA Oncol..

[219] Genelux Clinical Trial Summary. https://Genelux.Com/Clinical-Trials-Summary/.

[220] GL-ONC1 Administered Intravenously Prior to Surgery to Patients with Solid Organ Cancers. https://clinicaltrials.gov/study/NCT02714374.

[221] Expanded Access to Provide GL-ONC1 for the Treatment of Advanced Cancers with No Standard of Care. https://clinicaltrials.gov/study/NCT03420430.

[222] Safety and Efficacy of the ONCOlytic VIRus Armed for Local Chemotherapy, TG6002/5-FC, in Recurrent Glioblastoma Patients (ONCOVIRAC). https://www.clinicaltrials.gov/study/NCT03294486.

[223] Bendjama K., Cassier P., Moreno V., Doger B., Calvo E., de Miguel M., Jungels C., Erbs P., Carpentier D., Sadoun A. (2021). Abstract LB179: Oncolytic Virus TG6002 Locates to Tumors after Intravenous Infusion and Induces Tumor-Specific Expression of a Functional pro-Drug Activating Enzyme in Patients with Advanced Gastrointestinal Carcinomas. Cancer Res..

[224] West E.J., Sadoun A., Bendjama K., Erbs P., Smolenschi C., Cassier P.A., de Baere T., Sainte-Croix S., Brandely M., Melcher A.A. (2025). A Phase I Clinical Trial of Intrahepatic Artery Delivery of TG6002 in Combination with Oral 5-Fluorocytosine in Patients with Liver-Dominant Metastatic Colorectal Cancer. Clin. Cancer Res..

[225] Study of TBio-6517 Given Alone or in Combination with Pembrolizumab in Solid Tumors (RAPTOR). https://www.clinicaltrials.gov/study/NCT04301011.

[226] Chang C.-L., Ma B., Pang X., Wu T.-C., Hung C.-F. (2009). Treatment With Cyclooxygenase-2 Inhibitors Enables Repeated Administration of Vaccinia Virus for Control of Ovarian Cancer. Mol. Ther..

[227] Evgin L., Acuna S.A., de Souza C.T., Marguerie M., Lemay C.G., Ilkow C.S., Findlay C.S., Falls T., Parato K.A., Hanwell D. (2015). Complement Inhibition Prevents Oncolytic Vaccinia Virus Neutralization in Immune Humans and Cynomolgus Macaques. Mol. Ther..

[228] Lee N., Jeon Y.-H., Yoo J., Shin S., Lee S., Park M.-J., Jung B.-J., Hong Y.-K., Lee D.-S., Oh K. (2023). Generation of Novel Oncolytic Vaccinia Virus with Improved Intravenous Efficacy through Protection against Complement-Mediated Lysis and Evasion of Neutralization by Vaccinia Virus-Specific Antibodies. J. Immunother. Cancer.

